# Phylogenetic congruence, conflict and consilience between molecular and morphological data

**DOI:** 10.1186/s12862-023-02131-z

**Published:** 2023-07-05

**Authors:** Joseph N Keating, Russell J Garwood, Robert S Sansom

**Affiliations:** 1 Department of Earth and Environmental Sciences, University of Manchester, Manchester, M13 9PL UK; 2 School of Earth Sciences, University of Bristol, Life Sciences Building, Tyndall Avenue, Bristol, BS8 1TQ UK; 3 Natural History Museum, London, SW7 5BD UK

## Abstract

Morphology and molecules are important data sources for estimating evolutionary relationships. Modern studies often utilise morphological and molecular partitions alongside each other in combined analyses. However, the effect of combining phenomic and genomic partitions is unclear. This is exacerbated by their size imbalance, and conflict over the efficacy of different inference methods when using morphological characters. To systematically address the effect of topological incongruence, size imbalance, and tree inference methods, we conduct a meta-analysis of 32 combined (molecular + morphology) datasets across metazoa. Our results reveal that morphological-molecular topological incongruence is pervasive: these data partitions yield very different trees, irrespective of which method is used for morphology inference. Analysis of the combined data often yields unique trees that are not sampled by either partition individually, even with the inclusion of relatively small quantities of morphological characters. Differences between morphology inference methods in terms of resolution and congruence largely relate to consensus methods. Furthermore, stepping stone Bayes factor analyses reveal that morphological and molecular partitions are not consistently combinable, i.e. data partitions are not always best explained under a single evolutionary process. In light of these results, we advise that the congruence between morphological and molecular data partitions needs to be considered in combined analyses. Nonetheless, our results reveal that, for most datasets, morphology and molecules can, and should, be combined in order to best estimate evolutionary history and reveal hidden support for novel relationships. Studies that analyse only phenomic or genomic data in isolation are unlikely to provide the full evolutionary picture.

**Supplementary Information:**

The online version contains supplementary material available at 10.1186/s12862-023-02131-z.

## Introduction

Phylogenetic trees are integral to understanding evolution, yet the true tree is often unknown and must be estimated using phylogenetic data. The two main types of data used to reconstruct evolutionary relationships - genomic and phenomic - are usually studied in isolation, but increasingly they are combined. This offers some important advantages over partition-specific approaches, but also raises a series of important questions about not only the effect of combination, but also the evolutionary implications drawn. A crucial advantage is increased taxon sampling as terminals that yield only one type of data partition can be studied alongside each other. In this context, the development of Bayesian methods for incorporating fossil species as tips in clock analyses has proved particularly powerful [1, 2] given that fossil taxa generally only yield morphological characters and no molecular sequence data. Combining data partitions can result in synergy by revealing “hidden support” i.e. the support for some relationships increases, and/or unique relationships are recovered that are not resolved when either partition is analysed separately [3, 4]. However, despite these advantages and increasing prevalence, many questions remain regarding the efficacy of combining molecular and morphological data partitions. In this context we investigate the congruence between partitions, and the effects of combining them when using different partition sizes and inference methods, and the evolutionary implications drawn.

Morphological and molecular data partitions are frequently found to be highly incongruent [5–7]. Despite this, there has been very little study of this incongruence within the context of interaction between partitions, and whether they can be combined. This is particularly important given the debate concerning the intrinsically unbalanced proportion of phylogenetic signal contained within molecular and morphological partitions. Some authors have suggested that the morphological signal may be ‘swamped’ by the larger molecular partition/s [8–10], while others have found that a relatively small number of morphological characters can have a major effect on topology estimation [5, 11, 12].

Incongruence between morphological and molecular partitions may reflect a disparity in our understanding of the processes underlying these data, leading to misspecification of models. Our understanding of molecular evolution is informed by knowledge of the biochemical properties of the molecules themselves, as well as empirical measurements derived from sequences (e.g. base frequencies), enabling us to apply sophisticated models that fit the properties of the data [13–18]. In contrast, relatively little is known about the process of morphological evolution. This is principally because morphological characters, unlike molecular sites, are not equivalent; states are not comparable across characters and thus do not necessarily share similar properties [10]. Consequently, methods for estimating phylogeny using morphology are much simpler and make more general assumptions about the properties of the data [19, 20]. Conventionally, morphology has been analysed using maximum parsimony: an optimisation criterion that follows the principles of Ockham’s Razor and, as such, optimises the tree so that the fewest character state transitions are required. More recently, probabilistic methods have been applied to morphology. These methods utilise Markov models to describe evolutionary transitions between character states. The most commonly applied is the Mk model [19]. A number of simulation studies have explored these contrasting approaches for analysing morphological data [21–26], with most favouring Bayesian implementation of the Mk model over parsimony estimation [21, 22, 24, 26]. Yet it is unclear how comparable these simulations are to real morphological evolution, or whether different inference methods yield materially different trees in empirical examples. Modifying the assumptions of the simulation procedure can have a dramatic effect on the performance of inference methods. For example, both Bayesian implementation of the Mk model and parsimony approaches perform poorly for data simulated under a model incorporating character selection [26].

Given these questions over incongruence between data partitions and the suitability of morphological inference methods, a better understanding of the behaviour of combined morphological and molecular data in phylogenetic analysis is vital. Here we conduct a series of experiments to determine the effect of integrating morphological and molecular data in real world data. Through meta-analysis of empirical datasets comprising both molecular and morphological partitions, we compare the outcomes of different inference methods of morphological data, and the effects of analysing morphological data and molecular data individually or in concert. As such we test the following hypotheses:

1. Molecular and morphological partitions are combinable (i.e. both data partitions are best explained under a linked topology model, see methods for details).
2. Morphological trees are equally congruent with molecular trees, irrespective of the inference method used.
3. Analyses combining morphological and molecular data yield topologies that are different from those of individual analyses of molecular or morphological data (i.e. they occupy unique tree space).
4. There is a significant positive correlation between the proportion of morphological characters in combined datasets and the distance between combined- and molecular-only topologies.

By testing these hypotheses we critically consider the justification for combined analyses of morphology and molecules, and compare the evolutionary inferences drawn from combined vs. partition specific approaches to shed light upon the impact and utility of morphology in its own right as a source of phylogenetic data. We take a meta-analysis approach by sampling modern groups widely from across the tree of life from different authors in order to test the combinability and interaction of morphological and molecular partitions. This is a necessary first step before it is possible to consider the role and impact of fossil taxa in the context of combinability, given their intrinsic incompleteness and lack of molecular data.

## Methods

### Data selection and partitioning

Our data sample comprises previously published phylogenetic analyses with both molecular and morphological character partitions. Over 100 phylogenetic datasets were surveyed from previously published literature. For inclusion in this meta-analysis, datasets were required to have the following properties: (i) a minimum quantity of molecular data (parsimony-informative characters at least 10 times the number of taxa, sequences from a minimum of three genes); (ii) published and available sequence alignments with partition information; (iii) minimum of 10 taxa following editing (see below); (iv) minimum quantities of morphological data (informative characters at least 1.5 times the number of taxa); (v) minimal taxonomic overlap between datasets (less than 50% taxonomic overlap with any other dataset). If two matrices had taxon overlap greater than 50%, the most recently published matrix was selected. To ensure balance in the distribution of missing data, datasets were edited to remove taxa that lacked one of either the molecular or morphological partition and fossil taxa were thus removed [cf. 27]. Finally, datasets were discarded if any of the component partitions failed to achieve convergence when analysed independently. Failure to converge indicates that the posterior sample did not reach equilibrium and thus parameter estimates drawn from the posterior distribution should be interpreted as unreliable. Given the uneven distribution and inconsistent approach to identification and inclusion of autapomorphic and invariant morphological characters between studies, we chose to remove parsimony uninformative morphological characters and use the parsimony informative ascertainment bias model implemented in MrBayes [28]. All clock models, calibrations and constraints were removed to ensure equivalency between datasets in the meta-analysis. Molecular data were analysed using transition models and partitions as specified in the original published analyses, if present. Otherwise, the best fitting models, given the gene partitions and alignment of the original authors, were selected using PartitionFinder 2.1.1 [29] using the following specifications: model = aicc; MrBayes models only; schemes = greedy. All modified data matrices used in this study are available on Zenodo (DOI 10.5281/zenodo.6579584).

### Phylogenetic analysis

For Bayesian estimation of both morphological and molecular partitions, we used MrBayes version 3.2.6 [28]. We used 2 runs of 4 chains and sampled 10,000 trees, of which 25% were discarded as burnin. Convergence was assessed using Tracer 1.7, which is the most commonly used method for assessing convergence of Bayesian phylogenetic analyses [30]. Analyses were considered to have converged if the ESS scores of parameter estimates from independent runs were all greater than 200 and the traces of the independent runs were observed to have reached stationarity. The number of generations required to achieve convergence varied with each dataset (see supplementary datafiles on Zenodo 10.5281/zenodo.6579584). For parsimony analyses of morphological data (both equal and implied weighting), we used TNT version 1.5 [31]. We used ‘new technology’ searches with tree-drifting, tree-fusing, and sectorial searches (xmult: level 10) and subsequent branch breaking (bbreak) retaining a maximum of 100,000 MPTs for each matrix. In addition to equal weights (EW) parsimony searches, we also conducted implied weighting parsimony analyses (IW) using k = 3. This value was selected as it enforces strong weighting, is widely used, and is the default in TNT. We sampled 10,000 bootstrap replicates for parsimony searches using the TNT command ‘resample’. The results of all phylogenetic analyses conducted as part of this study are available on Zenodo (10.5281/zenodo.6579584).

### Bayes factor combinability test

The Bayes factor combinability test [32, 33] was applied to the results of Bayesian estimation of our combined datasets. This test compares the marginal likelihoods of two competing models: Model 1 (M1) assumes that branch lengths and tree topologies are independent between partitions; Model 2 (M2) assumes only independent branch lengths. Marginal likelihoods were estimated using stepping stone analysis implemented in MrBayes [28]. M1 has more free parameters than M2 and, as such, should be expected to better fit the data. A Bayes factor of 3–5 log units is interpreted as strong evidence in support of one model over another, while a Bayes factor of 5 log units or greater is interpreted as very strong evidence [34]. Thus, if the marginal likelihood of M1 is 5 log units or greater than M2, we interpret this as very strong evidence of incongruence between partitions (i.e. the data are uncombinable). On the other hand, if M1 and M2 are less than 3 log units different, it suggests that there is little evidence of incongruence. Consequently we should favour the combined model with fewest free parameters (M2). Convergence of stepping stone analyses was assessed by comparison of the marginal likelihood estimates from independent runs. Analyses were considered converged if the standard deviation of independent marginal likelihood estimates was < = 5 log units.

### Measuring topological congruence

We compared topologies using Robinson-Foulds (RF) and quartet distances [35, 36]. These were normalised by dividing distances by the sum of resolved bipartitions/quartets across both trees (i.e. maximum distance). In addition to these symmetric measures, we applied an asymmetric measure in order to compare the proportional congruence (with molecular data) of trees estimated using different morphological inference methods. We define proportional congruence as the number of bipartitions/quartet statements (i.e. relationships) shared between a partially or fully resolved query tree (morphological) and a fully resolved reference tree (molecular, see below), expressed as a proportion of the total number of comparable bipartitions/quartet statements present in the query tree. Thus if the query (morphological) tree has five bipartitions, the reference (molecular) tree has 10 bipartitions and both share four bipartitions, the query tree has a proportional congruence of 0.8 with the reference tree (it has 5 bipartitions that can be compared with the fully resolved reference tree, four of which are congruent). The reference tree has 4 unique bipartitions that are not resolved in, but are nonetheless compatible with, the query tree. This measure of congruence was used because it produces values that can be compared across datasets with different numbers of taxa, and it allows for the congruence of morphological consensus trees with different resolutions to be assessed. Proportional congruence was measured with a custom R script (SI) using the Quartet [37], Ape [38] and Phangorn [39] packages (see SI).

We obtained standard consensus trees for each morphological inference method: most parsimonious trees estimated using equal or implied weighting were summarised using a strict consensus tree; Bayesian morphological posterior trees were summarised using a 50% majority rule consensus tree. We calculated proportional congruence for each standard morphological consensus tree per dataset with a fully-resolved molecular reference tree. For the reference tree, we used the molecular-only maximum clade credibility tree (i.e. a single fully resolved tree within the posterior distribution containing the maximum sum of posterior probabilities across each clade). We also measured the proportional congruence with the molecular maximum clade credibility tree using a broader range of non-standard morphological consensus trees obtained via collapsing nodes under a certain threshold of support: For Bayesian inference, we obtained the all-compatible-consensus tree using the ‘sumt Contype = Allcompat’ command in MrBayes. We then collapsed nodes with less than x posterior probability (where x = 0, 0.01, 0.02, 0.03… 0.99). For parsimony (both equal and implied weights), we obtained the strict consensus tree and collapsed nodes with less than x bootstrap support (where x = 0, 1, 2, 3… 99). These methods are analogous in that a highly resolved tree is iteratively collapsed based on support values. Hence we are able to test if differences in proportional congruence of morphological inference methods are correlated with resolution, irrespective of inference method or ‘standard’ consensus method. In a further attempt to eliminate consensus method as a factor, we compared the proportional congruence (with the molecular maximum clade credibility tree) of fully resolved optimal trees inferred from morphological data (i.e. most parsimonious trees or maximum clade credibility trees). Results were plotted using the package ggplot2 [40].

### Tree space visualisation

Tree space was visualised in R through a custom function using the phylogenetic packages Phangorn; Ape; and Quartet; and the parallel packages Foreach [41]; and doMC [42]. For computational efficiency we randomly sampled 1000 post-burnin trees from the Bayesian posterior distribution of the morphology-only analysis, the molecular-only analysis, and the combined analysis. We also randomly sampled 1000 most parsimonious trees if the number of most parsimonious trees exceeded 1000, or all of the most parsimonious trees if there were fewer than 1000. All trees were unrooted. We produced distance matrices between each tree in the sample using Robinson-Foulds distance [35] and Quartet distance [36]. We used classical multidimensional scaling to reduce the dimensions of the distance matrix into 2 axes. The results were plotted using the package ggplot2 [40]. The custom R script used to conduct tree space analysis, as well all distances matrices and tabulated eigenvalues are available on Zenodo (10.5281/zenodo.6579584).

## Results

A total of 32 datasets fulfilled our strict selection criteria. These include 14 vertebrate datasets: Tetraodontiformes [43]; Ostariophysi [44]; Mammalia [45]; Lemuriformes [46]; Sphenisciformes [47]; Osteoglossiformes [48]; Mysticeti [49]; Squamata [50]; Serpentes [51]; Cetacea [52]; Chiroptera [53]; Caviidae [54]; Abrotrichini [55]; Actinopterygii [56], 14 arthropod datasets: Hemiptera [57]; Hymenoptera [1]; Arthropoda [58]; Palpimanoidea [59]; Formicidae [60]; Opiliones [61]; Malacostraca [62]; Stygnopsidae [63]; Hydrophilidae [64]; Tribelocephalinae [65]; Apinae [66]; Biblidinae [67]; Hydroptilidae [68]; Nephilidae [69]; 1 mollusc dataset: Mollusca [70], 1 annelid dataset: Fabriciidae [71], 1 brachiopod dataset: Rhynchonellida [72] and 1 cnidarian dataset: Hexactinellida [73]. Together, these datasets comprise a total of 1,137 taxa, 8,197 parsimony informative morphological characters and 95,107 parsimony informative molecular characters.

### Combinability of morphological and molecular data partitions

Bayes factor analysis was possible for 20 of our 32 combined datasets (12 dataset sets reached 1,000,000,000 generations without M1 and/or M2 converging, and were thus disregarded). Of the 20 fully converged analyses, 6 datasets showed strong (Bayes factor > 3) or very strong (Bayes Factor > 5) support for partition uncombinability (i.e. the marginal likelihood of M2 was 3 or more log units greater than the marginal likelihood of M1), while 14 datasets supported partition combinability (i.e. the marginal likelihood of M2 was less than 3 or more log units greater than the marginal likelihood of M1). These results indicate that, for the majority of combined datasets in our study (70%, n = 20), molecular and morphological data partitions are best explained under a single evolutionary process. Consequently, we tentatively accept hypothesis 1 i.e. that molecular and morphological partitions are combinable.

### Topological congruence of molecular and morphological consensus trees under different inference methods

Standard consensus trees obtained using different methods of morphological estimation differ significantly in their congruence with topologies estimated from corresponding molecular data (supplementary Figs. 1, 2); strict consensus trees of equal weighting parsimony searches and 50% majority rule consensus trees from Bayesian searches both exhibit greater congruence with molecular trees than strict consensus trees from implied weighting searches (ANOVA with repeated measures, p = 0.012 for both proportion of bipartitions and quartet metrics, with post-hoc pairwise tests). Our results therefore lead us to reject hypothesis 2: Topologies obtained using different methods of morphological estimation **do** vary in respect to their congruence with topologies estimated from independent molecular data. However, a lot of the variation is due to the standard methods of consensus estimation and their concomitant levels of resolution [25, 26].

**Fig. 1:**
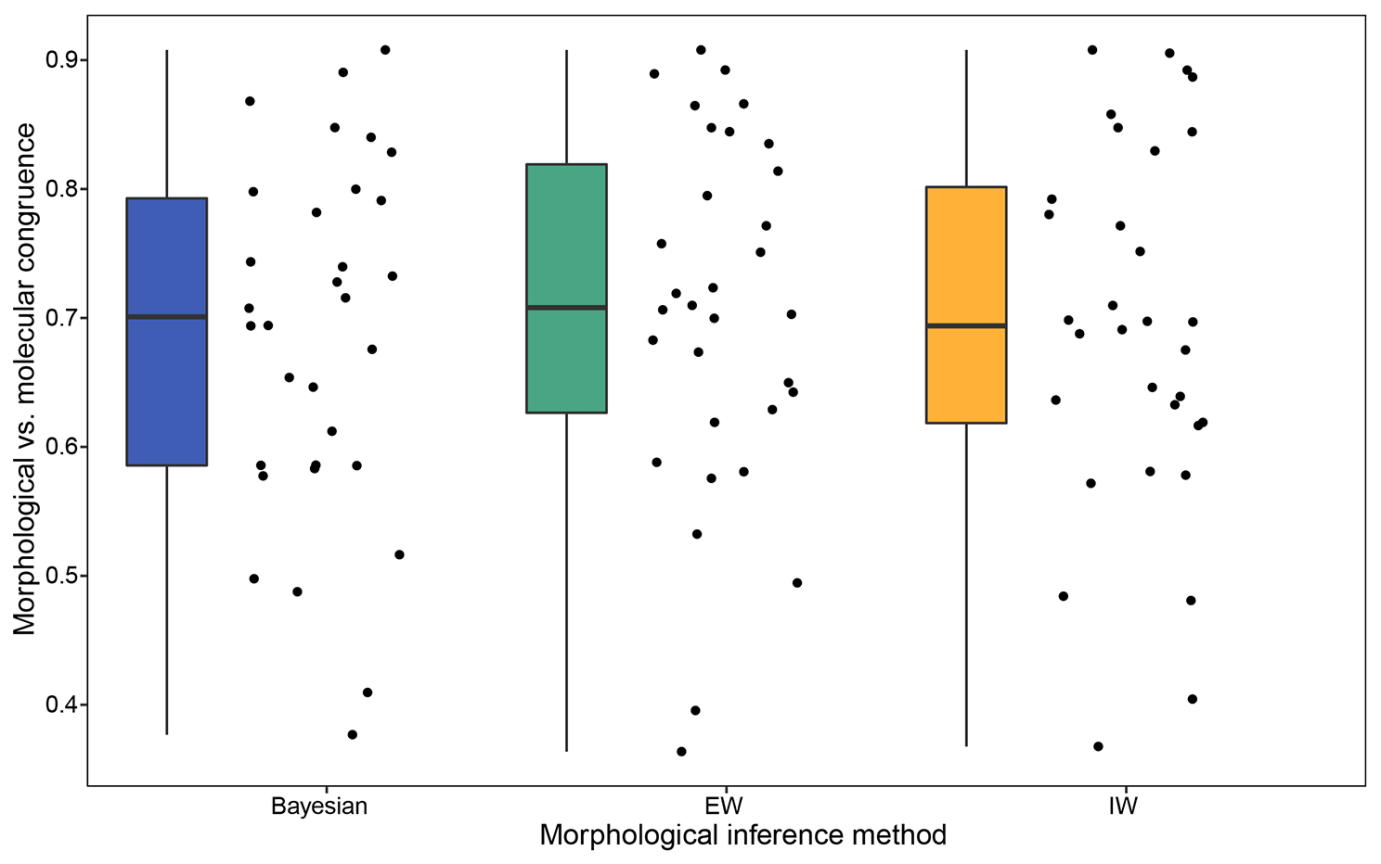
Optimal morphological trees (i.e. most parsimonious trees and Bayesian maximum clade credibility trees) have similar congruence with the corresponding molecular trees (p = 0.167, ANOVA with repeated measures). Congruence is measured using the mean proportion of quartet statements that morphological trees share with molecular-only maximum clade credibility tree. The 32 points represent mean proportional congruence between the morphological and molecular trees for each inference method, per dataset

To further investigate the effect of tree resolution we took two approaches. Firstly, we compared fully-resolved optimal trees estimated using different morphological inference methods. For each dataset, we calculated the mean proportional congruence (see methods) of most parsimonious trees estimated under equal vs. implied weighting and compared this with the proportional congruence of the maximum clade credibility tree from the morphology-only Bayesian posterior distribution (Fig. 1, supplementary Fig. 3.). We found no significant difference in terms of proportional congruence of optimal trees (ANOVA with repeated measures, p = 0.821/0.167 for proportion of bipartitions and quartet metrics respectively). Secondly, by collapsing nodes iteratively based on support values (posterior probability or bootstrap support), we find that Bayesian and parsimony consensus trees show similar relationships between congruence, resolution and morphological tree support (Supplementary Figs. 4–7). Most datasets exhibit a negative correlation between proportional congruence and tree resolution, and a positive correlation between proportional congruence and overall tree support: at higher node collapsing thresholds, trees tend to be less resolved, have higher overall support and exhibit higher proportional congruence. If there were no relationship between congruence and resolution/support, we would not expect to observe a trend towards higher proportional congruence (except in extreme cases such as a morphological tree with a single node, which by definition must be either 100% congruent or 0% congruent with the molecular tree). Importantly, we find that Bayesian and parsimony methods follow similar trajectories in these plots. This means that morphological trees of similar resolution exhibit similar congruence with corresponding molecular trees, irrespective of optimisation criteria. Our results thus suggest that differences in proportional congruence between standard morphological consensus trees and corresponding molecular trees can largely be explained by the differences in the resolution of the morphological consensus trees.

### Tree space sampling and visualisation of individual and combined datasets

**Fig. 2:**
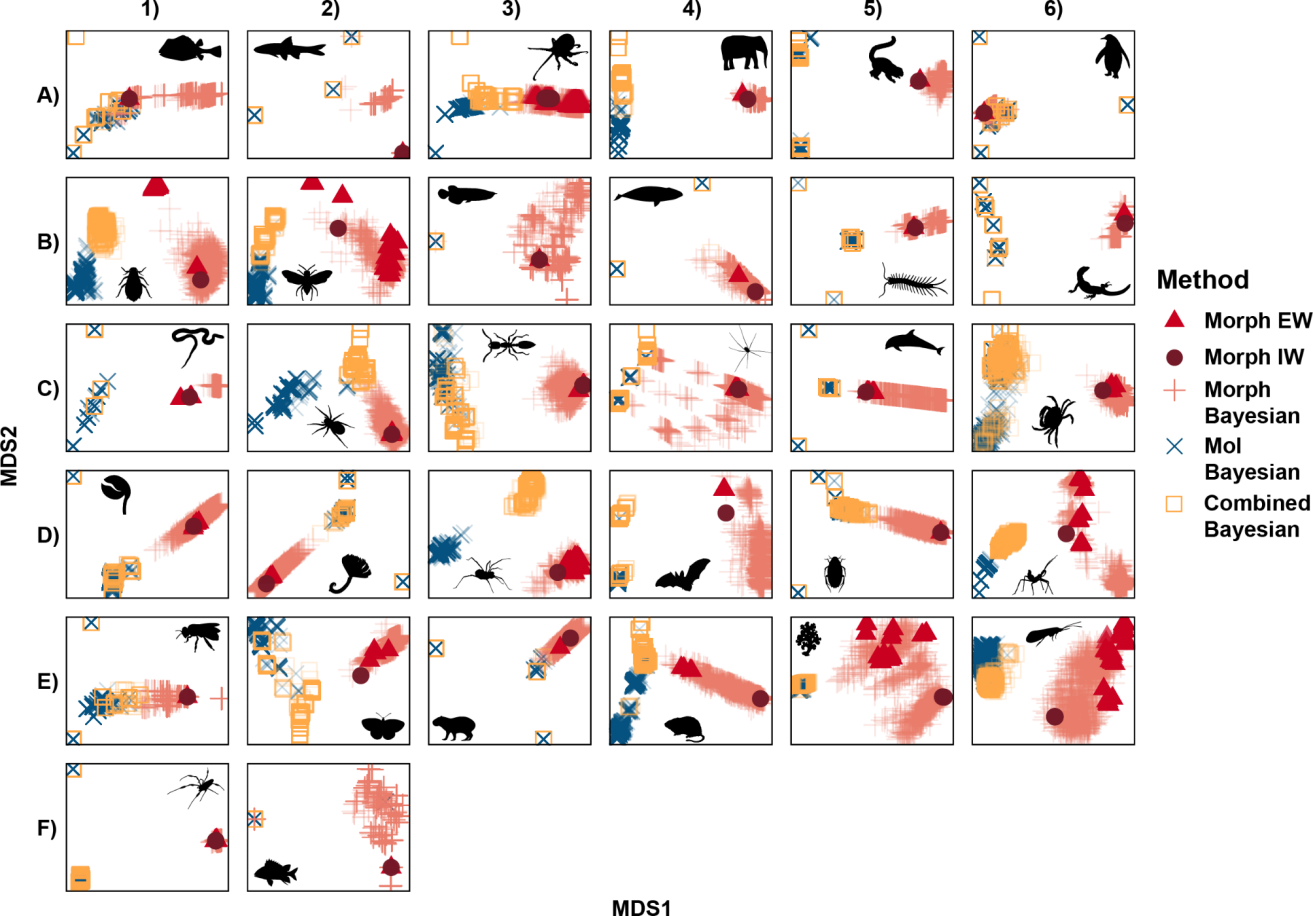
Treespace visualization of 32 empirical datasets using the quartet distance metric. Visualizations show Bayesian molecular-only posterior trees (blue crosses); Bayesian combined morphology and molecular posterior trees (open orange squares); Bayesian morphology only posterior trees (pink crosses); equal weighting most parsimonious trees (red triangles) and implied weighting most parsimonious trees (dark red circles). Trees sampled using various morphological methods tend to cluster together; molecular and combined trees tend to be more similar than molecular and morphology trees, but combined trees seldom completely overlap with molecular-only trees; combined analyses thus sample unique areas of treespace. (**A1**) Tetraodontiformes; (**A2**) Ostariophysi; (**A3**) Mollusca; (**A4**) Mammalia; (**A5**) Lemuriformes; (**A6**) Sphenisciformes; (**B1**) Hemiptera; (**B2**) Hymenoptera; (**B3**) Osteoglossiformes; (**B4**) Mysticeti; (**B5**) Arthropoda; (**B6**) Squamata; (**C1**) Serpentes; (**C2**) Palpimanoidea; (**C3**) Formicidae; (**C4**) Opiliones; (**C5**) Cetacea; (**C6**) Malacostraca; (**D1**) Rhynchonellida; (**D2**) Fabriciidae; (**D3**) Stygnopsidae; (**D4**) Chiroptera; (**D5**) Hydrophilidae; (**D6**) Tribelocephalinae; (**E1**) Apinae; (**E2**) Biblidinae; (**E3**) Caviidae; (**E4**) Abrotrichini; (**E5**) Hexactinellida; (**E6**) Hydroptilidae; (**F1**) Nephilidae; (**F2**) Actinopterygii. Silhouettes for **C4**, **D3** and **E6** were created by Gareth Monger, Jennifer Trimble and JCGiron respectively and are reproduced here under the CC BY 3.0 licence

Tree space visualisations (Fig. 2, supplementary Fig. 8) reveal that morphological- and molecular-only analyses tend to sample mutually exclusive areas of tree space. In contrast, the different methods of morphological-only analyses typically sample overlapping regions of tree space with varying levels of precision. Implied weighting parsimony is the most precise. In other words, the most parsimonious trees under implied weighting parsimony tend to be very similar to one another and thus occupy a small region of tree space (Fig. 2, supplementary Fig. 8). On the other hand, Bayesian inference is the least precise; Bayesian posterior trees tend to be fairly different from one another and occupy a diffuse area of tree space. Combined molecular and morphological analyses tend to be more similar to the molecular-only trees than to any of the morphological-only trees, suggesting that the molecular partition provides most of the phylogenetic signal in combined analyses. A typical example is the stygnopsid harvestman dataset (Fig. 2D3) for which there is clear separation between the morphological-only and molecular-only trees on the first axis, whilst combined dataset trees are separate from both on the second axis. The percentage of variance explained by the first two principal coordinates is < 50% for 24 out of 32 datasets when analysed using the quartet metric, and 29 out of 32 datasets when analysed using the RF metric (Fig. 2, Supplementary Fig. 8). This indicates that data retain a high degree of dimensionality following multidimensional scaling. Nonetheless, in most datasets, the morphology-only trees, molecular-only trees, and combined dataset trees show separation along the first MDS axis, indicating that this is the most important source of variation between trees across datasets. Combined estimates rarely completely overlap molecular-only estimates, thus supporting hypothesis 3: combined analyses sample unique regions of tree space not explored by analysing the individual partitions separately. Supporting this observation, we find that 21 of 32 combined consensus trees possess at least 1 unique clade not present in either the Bayesian morphological-only or molecular-only consensus trees (supplementary Table 1). Thus, our results suggest analyses combining morphological and molecular data yield topologies that are different from those of individual analyses of molecular or morphological data.

### Relative partition size and phylogenetic signal

**Fig. 3:**
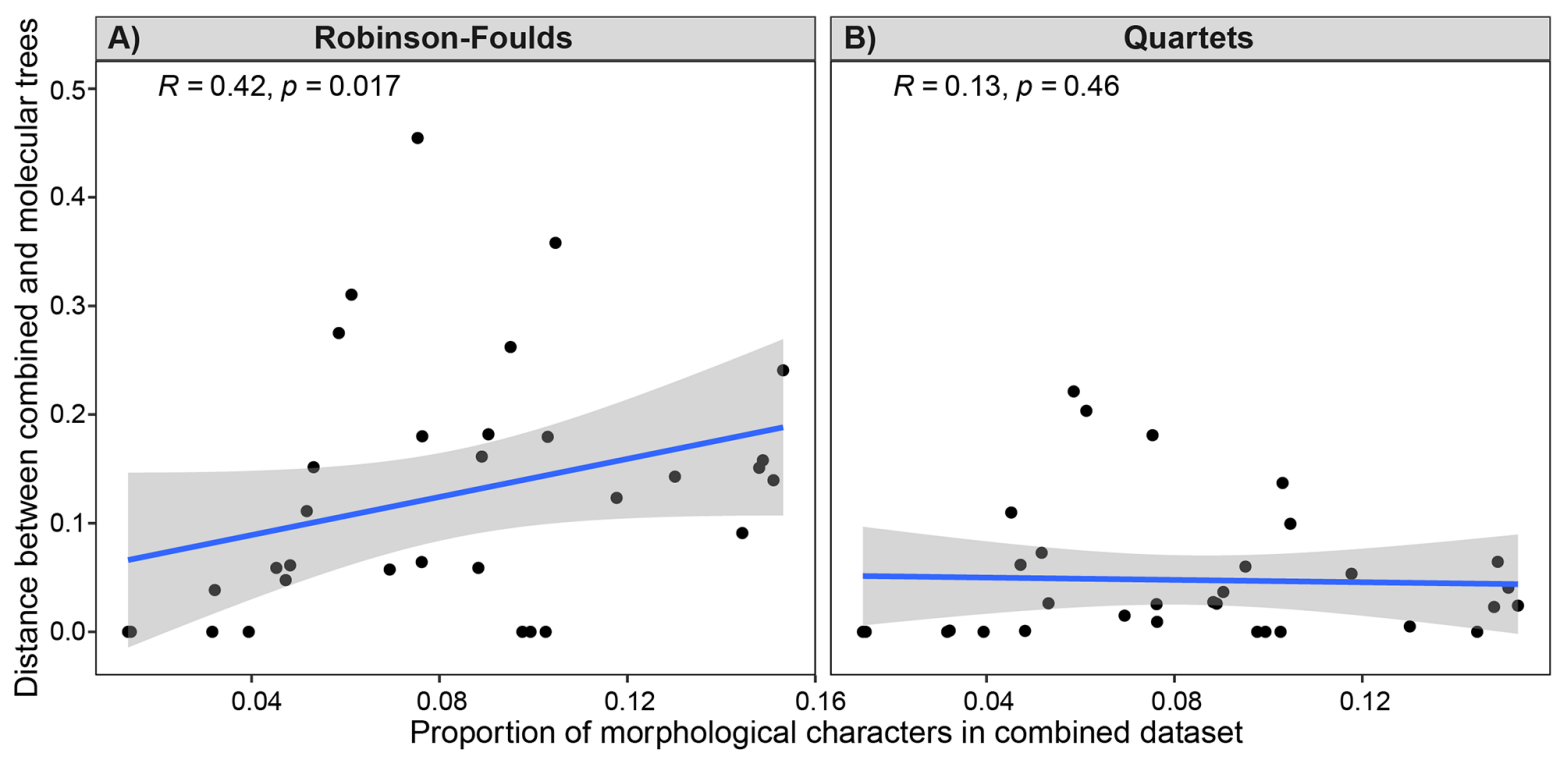
Relationship between the proportion of morphological characters in the combined dataset and the distance between combined- and molecular-only trees. (**A**) Robinson-Foulds distance between the combined 50% majority rule consensus tree and the corresponding molecular-only 50% majority rule consensus tree against proportion of morphological characters; (**B**) quartet distance between the combined 50% majority rule consensus tree and the corresponding molecular-only 50% majority rule consensus tree against proportion of morphological characters

To test the role of partition size imbalance, we compared datasets in terms of their relative partition sizes (i.e. the proportion of the total parsimony informative characters that are morphological vs. the distance between combined and molecular-only consensus trees). We find a significant positive correlation (R = 0.42, p = 0.017) between the proportion of morphological characters in the combined dataset and the RF distance between combined and molecular-only consensus trees: combined datasets that contain a higher proportion of parsimony informative morphological characters tend to produce consensus trees that are more distant to corresponding molecular-only consensus tree (Fig. 3A). However, we find no significant relationship (R = 0.23, ρ = 0.21) when congruence is calculated using resolved quartets (Fig. 3B). The discrepancy between quartet- and RF distances is likely due to the sensitivity of the latter to rogue taxa [74]. A single branch rearrangement can result in the maximum possible RF distance between two trees, while the same is not true for quartet distance. Due to this behaviour, quartet distance is generally regarded as a more robust tree distance measure than RF distance [25]. Our results suggest that trees inferred using a greater proportion of morphological characters are prone to rogue taxa, thus inflating the RF distance relative to the quartet distance. Based on the results using the Quartet distance, we reject hypothesis four: we do not find a significant positive correlation between the proportion of morphological characters and the distance between combined and molecular-only topologies.

## Discussion

### Molecular and morphological data are frequently consilient

Our results agree with previous studies [5, 6] that morphological and molecular data partitions often exhibit conflicting phylogenetic signals: analysing partitions separately typically results in the sampling of unique (non-overlapping) areas of tree space (Fig. 2, Supplementary Fig. 8). However, the results of our stepping stone analyses demonstrate that morphological and molecular data partitions are frequently combinable. For 14 of 20 datasets, the combined data are better explained by a common linked topology rather than partition-specific topologies. This indicates that conflict between partitions is insufficient to suggest that partitions were generated under independent evolutionary processes and can be analysed simultaneously in combined analysis (Supplementary Table 2).

### Incorporating uncertainty is more important than choice of morphological inference method

Congruence between morphological and molecular trees is highly dependent on the inference method applied to morphological data i.e. Bayesian versus parsimony searches. Bayesian morphology-only consensus trees are significantly more similar to corresponding molecular trees than are parsimony morphology-only consensus trees. This is, in large part, related to the resolution of the morphological consensus tree, and we find no difference in proportional congruence of optimal fully resolved trees (i.e. most parsimonious trees vs. Bayesian maximum clade credibility trees, Fig. 1, supplementary Fig. 3). It is perhaps not surprising that Bayesian and parsimony morphological consensus trees are not equally congruent with molecular trees given that these methods take fundamentally different approaches to the sampling then summarising of tree space [25, 75]. Parsimony methods use heuristic hill climbing algorithms that tend to sample one or a few optimal trees, typically summarised using a strict consensus tree. On the other hand, Bayesian analyses utilise a Markov chain Monte Carlo (MCMC) algorithm, which samples thousands of trees in proportion to their posterior probability. This posterior sample is typically summarised using a majority rule consensus, in which only nodes with ≥ 0.5 posterior probability are retained. By calculating bootstrap support for parsimony standard consensus trees and collapsing nodes iteratively based on their support, we are able to compare parsimony and Bayesian trees of similar support and resolution. For both Bayesian and parsimony trees, we find that as higher collapsing thresholds are applied, and the resolution of the resulting tree decreases, proportional congruence with the molecular maximum clade credibility tree increases. This suggests there may be a relationship between the morphological support for a bipartition and its congruence with the molecular tree: well supported nodes on morphological trees are more likely to be congruent with molecular trees, whereas poorly supported nodes on morphological trees are less likely to be congruent with molecular trees. This hints at deep consilience between morphological and molecular data. Previous discourse on the accuracy of parsimony versus Bayesian methods of inference from morphological data have been intrinsically linked to debates about relative precision and comparability of mode of consensus [22, 24–26, 75–77]. We demonstrate that differences are largely negated when consensus methods of equivalent precision are applied (Supplementary Figs. 4–7), or else when optimal fully resolved trees are considered (Fig. 1).

### Combining molecular and morphological partitions finds unique trees and unique relationships

Combined analyses frequently sample unique regions of tree space not recovered when morphological and molecular partitions are analysed individually (tree space visualisations, Fig. 2, Supplementary Fig. 8). Furthermore, we find that 21 of 32 combined consensus trees possess at least 1 bipartition that is not present in either the molecular-only or morphological-only Bayesian consensus tree (Supplementary Table 1). As such, our meta-analysis strongly supports the notion that combining morphological and molecular partitions can yield hidden support for novel clades [78, 79]. Hidden support occurs when a phylogenetic signal shared between partitions is amplified via combining partitions and dispersing conflicting signals [4]. This is beneficial if the amplified phylogenetic signal is consistent with the true tree. However, combining partitions can also lead to dispersing the true phylogenetic signal and amplifying homoplastic signals. In such a scenario, novel clades unique to the combined analysis would be inconsistent with the true tree. Our Bayes factor stepping stone analyses (Supplementary Table 2) demonstrate that morphological and molecular partitions are frequently combinable, even though topological conflict arises when partitions are analysed separately (tree space visualisation, Fig. 2, Supplementary Fig. 8). These results lend support to the idea that novel clades resulting from analysis of combined datasets are consistent with the true tree, rather than merely an artefact of homoplasy amplification. Such synergistic interaction of data partitions is one of the key proposed advantages of combined analyses vs. consensus-based approaches to data combination [4, 78]. Although we demonstrate that partitions are generally combinable, this is not always the case (6 of 20 datasets in our metaanalysis are uncombinable). We recommend that combinability tests [32, 33] and tree space visualisation [75, 80, 81] should be conducted on morphological and molecular partitions prior to combined analysis. Topologies should be interpreted in light of the combinability of partitions these analyses reveal. The insight provided by these analyses are essential for interpreting novel clades in combined consensus trees that are not resolved when analysing morphological and molecular partitions separately.

### Phylogenetic signal strength is not related to partition size

We do not find a significant relationship between the proportion of morphological data in an analysis and the quartet distance between the combined and molecular-only consensus trees (Fig. 3). Previous studies have debated the extent to which morphological signal is ‘swamped’ by larger molecular partitions [5, 8–12]. The notion that a small number of morphological characters could alter the topology inferred from a substantially larger molecular partition is supported by the fact that molecular genomic topologies, which include tens of thousands of sites, can be extremely sensitive to character inclusion, such that removal of a very small percentage of molecular sites is sufficient to drastically alter topological inference [82]. Our results suggest that the relative influence of the phylogenetic signal contained within molecular and morphological partitions is not merely a product of their relative size. Combined trees from datasets with a small proportion of morphological characters can exhibit extensive incongruence with the molecular-only topology and vice versa. Data ‘swamping’ by larger molecular partitions may well be a problem within datasets, as increasingly more comprehensive genomic sequences are combined with a finite number of morphological characters. Indeed, some morphological datasets with extensive homoplasy and/or conflicting signals may be more susceptible to swamping. However, there is no evidence that swamping produces a general pattern across datasets, and as such, there is unlikely to be a single optimal ratio between molecular and morphological characters that can be applied to all combined analyses. The degree to which the combined tree is congruent with the molecular-only tree is highly dependent on the dataset. This likely reflects intrinsic differences between data partitions such as the distribution of evolutionary rates among characters and lineages, the mode/selectivity of evolution, and the appropriateness of the specified evolutionary model.

### Evolutionary and conceptual differences underlie incongruence between morphological and molecular partitions

Given the differences between molecular and morphological partitions observed in empirical datasets, it is necessary to consider phenomena that could account for this pattern. Firstly, incongruence between molecular and morphological data could reflect real evolutionary differences between these partitions. Molecular and morphological partitions are likely subjected to different levels of ecological, developmental or functional selective constraints. These could manifest in differences in data properties, and concomitant varying patterns of: (1) homoplasy; (2) rate heterogeneity; (3) character integration / non-independence; and (4) incomplete lineage sorting. Many of these data properties have been explicitly considered in the context of molecules versus morphology [e.g. 26, 27, 83–86]. Alternatively, incongruence may be a consequence of the inherently different ways that the two partitions are conceptualised and treated, in particular: (1) data sampling and (2) inference method adequacy. Choice of genes or morphological characters may explain some of the incongruence between molecular and morphological partitions, as could fundamental differences in the objectivity and approach to character definition. Yet, in our meta-analysis, we find incongruence between these partitions is fairly ubiquitous, regardless of data set.

There has been extensive debate with respect to model adequacy concerning the suitability of parsimony and likelihood methods for inferring of phylogenies from morphological data. However, directly comparing these methods is difficult because - in contrast to the assumptions of likelihood-based models, such as the Mk model, which are explicit - the assumptions of parsimony are implicit and often unintuitive [20, 87]. The standard Mk model assumes stationarity, that is, that each state occurs in a phenotype with equal frequency, and that transitions between states are at equilibrium, thus the frequency of states remains approximately constant throughout evolutionary time [88]. It also assumes symmetry between state transitions, that is, that the probability of changing from state A to state B is the same as changing from state B to state A. Contrary to these assumptions, morphology is thought to evolve via adaptation and directional selection [89, 90], which is consistent with a nonstationary process in which state frequencies are unequal and change through time [91]. Furthermore, empirical morphological characters frequently exhibit state distributions that are consistent with transition rate asymmetry (e.g. Dollo Characters [e.g. Dollo characters, 92, 93]). Parsimony does not assume stationarity [94] or equal rates between state transitions [95, 96], but it is generally agreed that parsimony methods assume characters are independent [20]. In reality, many morphological characters are highly non-independent due to developmental or functional linkage and hierarchical nesting [84, 97–100]. Indeed, sub-partitions of morphological data have been found to have significant differences e.g. soft tissue characters and osteological characters [101, 102], dental characters and osteological characters [27], cranial and postcranial characters [103], and appendage and non-appendage characters [104]. As such, a key focus for future work in morphological phylogenetics is to develop new models that incorporate these observed empirical properties of morphological character evolution.

### The inclusion of fossils and development of morphological models may increase congruence

Looking to the future, two other factors may help bridge the observed gap between morphology and molecules (1) the inclusion of fossil taxa, (2) development of new models.

Including fossils in total evidence analyses has numerous implications: Increased taxon sampling is generally considered to improve the accuracy of phylogenetic estimation [105, 106], and fossil taxa in particular have been shown to possess greater topological influence than living taxa [107, 108]. They ameliorate the over-precision of some inference methods [109]. Fossils provide information about character polarity and can break long branches by populating stem groups, thus helping to mitigate topological biases affecting deeply diverging extant lineages [108, 110, 111]. Indeed, a number of studies have shown that including fossils within morphological datasets improves their congruence with molecular trees [111–113]. Fossils also provide stratigraphic information that can inform topology under total-evidence clock analyses which incorporate fossil taxa as tips [1, 2]. Incorporating stratigraphic data can have a dramatic impact on morphological topology estimation [114, 115], allow the inference of more accurate trees [109, but see also 116], and can help reconcile incongruent evolutionary timescales inferred from fossils vs. molecular clocks [117]. However, quantifying the impact of including fossils in combined analyses necessitates a more holistic understanding, including the effect of a number of variables, namely: (1) Increased taxon sampling; (2) Uneven and non-random patterns of missing characters data and taxa; (3) Non-clock vs. clock models. Disentangling these complex and interrelated variables is impossible without first quantifying patterns of incongruence in extant taxa, the only source for which molecular and morphological data are both available. The results of our analyses will thus provide an important benchmark for future studies aiming to characterise the effect of fossils in combined analyses.

In addition to the inclusion of fossils, the development and refinement of morphological models holds great potential to improve phylogenetic estimates from discrete character data. Molecular substitution models have undergone extensive development over the last 50 years, allowing for more complex, biologically realistic models accommodating the heterogeneous nature of molecular evolution [20, 118]. In contrast, the development of morphological models has only just begun. Early improvements have relaxed of a number of problematic assumptions of the Mk model, allowing for asymmetric state changes [93], unequal state frequencies [33], nonstationarity [91] and character nonindependence [98]. In particular, the use of structured Markov models equipped with hidden states provides a promising framework to model the nonindependence of characters due to hierarchical contingencies, developmental linkage and/or serial homology [98, 99, 119, 120]. Application of more nuanced and appropriate models of morphological evolution, together with incorporation of fossil taxa, will undoubtedly improve the accuracy of morphological phylogenetic estimation. As both morphological and molecular models improve, we should expect both to converge upon the true tree, thus congruence between these partitions should increase. We therefore expect that combined analysis (including total-evidence analysis of living and fossil taxa) will become an increasingly important tool for resolving relationships and understanding consilience and conflict between morphological and molecular data.

## Conclusion

Phylogenetic estimation using both molecules and morphology offers a number of advantages over separate analyses, including improved taxon sampling and the ability to reveal hidden support. However, there is little understanding of how these different data partitions interact. Our results show that, when analysed separately, molecular and morphological partitions of combined datasets often yield very different trees. This is true irrespective of the inference method used to analyse morphology. Analysing the combined dataset often results in the sampling of unique areas of tree space. Our results underscore the importance of morphology; even small quantities of morphological data relative to molecular data can result in sampling unique trees. Furthermore, our Bayes Factor analyses reveal that morphological and molecular partitions are often compatible. The topologies resulting from each partition are not so different from each other as to indicate that they result from different underlying evolutionary processes. On the basis of our results, we recommend combining partitions where possible because it enables recovery of novel clades, potentially consistent with the true tree, that would otherwise remain hidden. However, consideration of the ‘combinability’ of morphological and molecular data partitions is essential for interpretation of novel topologies and hidden support. Incorporation of fossil taxa, as well as development of more nuanced and sophisticated models of morphological evolution, will undoubtedly improve tree estimation and help bring parity between morphology and molecules. In all cases, the differences found between morphological and molecular partitions indicate that studies focusing on just one class of data will be getting an incomplete picture as to the relationships and evolution of the group in question. Morphology continues to be essential, not only because it is the only way to incorporate fossil taxa in phylogenies, but also because of the intrinsic value it has for reconstructing relationships.

## Electronic supplementary material

Below is the link to the electronic supplementary material.

Supplementary Material 1

## Data Availability

The data supporting the findings reported in this paper are openly available from the Zenodo repository at 10.5281/zenodo.6579584.
