# Supplementary material for: Phylogenetic congruence, conflict and consilience between molecular and morphological data
Source: BMC Ecol Evol. 2023 Jul 5;23:30. doi: 10.1186/s12862-023-02131-z (PMC10321016; doi:10.1186/s12862-023-02131-z)
Supplement: Supplementary file 1 — Supplementary Material 1 [file 12862_2023_2131_MOESM1_ESM.docx]

# Supplementary Information


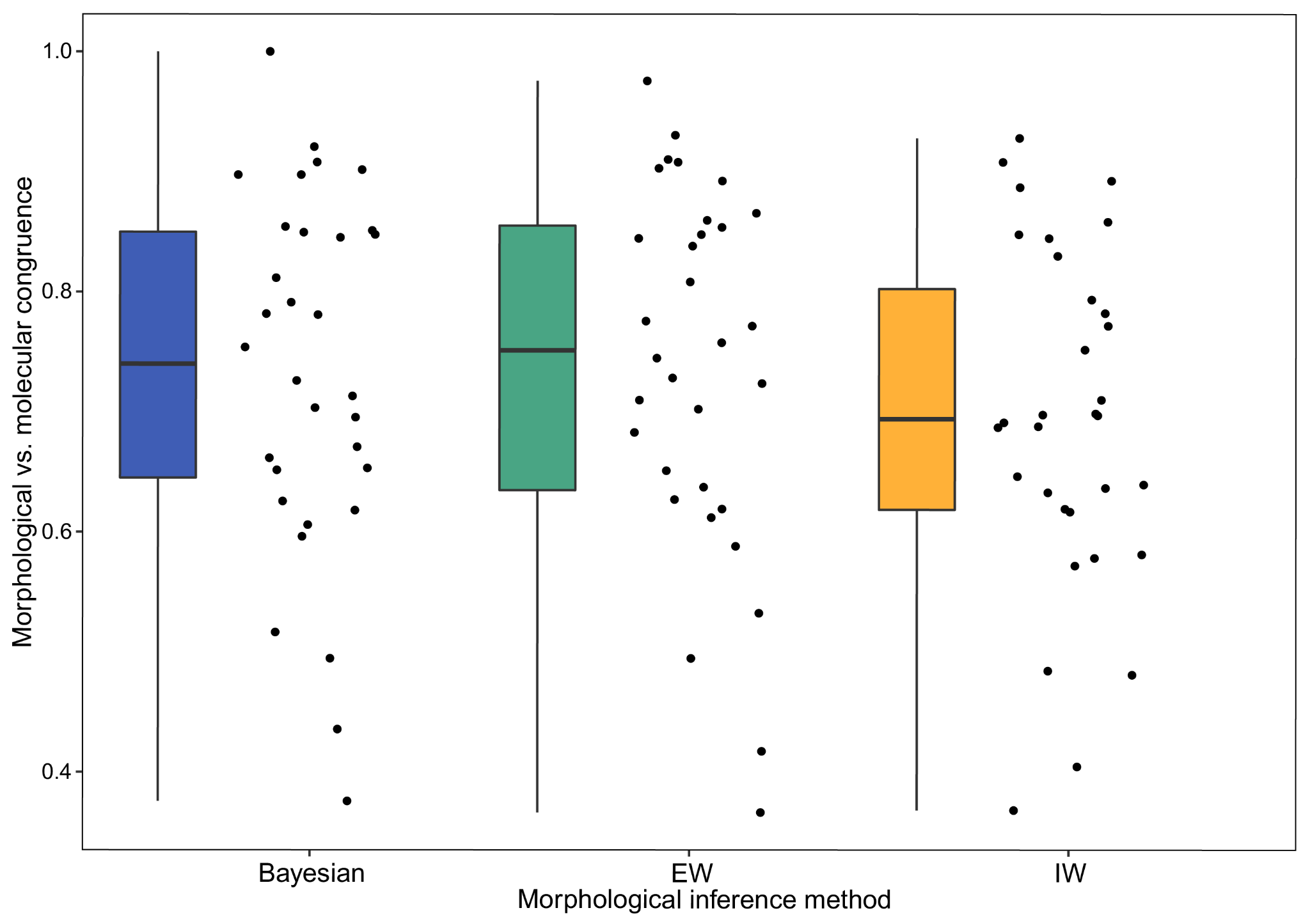


*Supplementary Fig. 1. Parsimony strict consensus trees and Bayesian majority rule consensus trees estimated from morphological data have significantly different congruence with molecular trees (p = 0.012, ANOVA with repeated measures). Congruence is measured using the proportion of quartet statements shared with the molecular-only maximum clade credibility tree. Points represent proportional congruence of each morphological consensus tree (with the corresponding molecular maximum clade credibility tree) per dataset.*


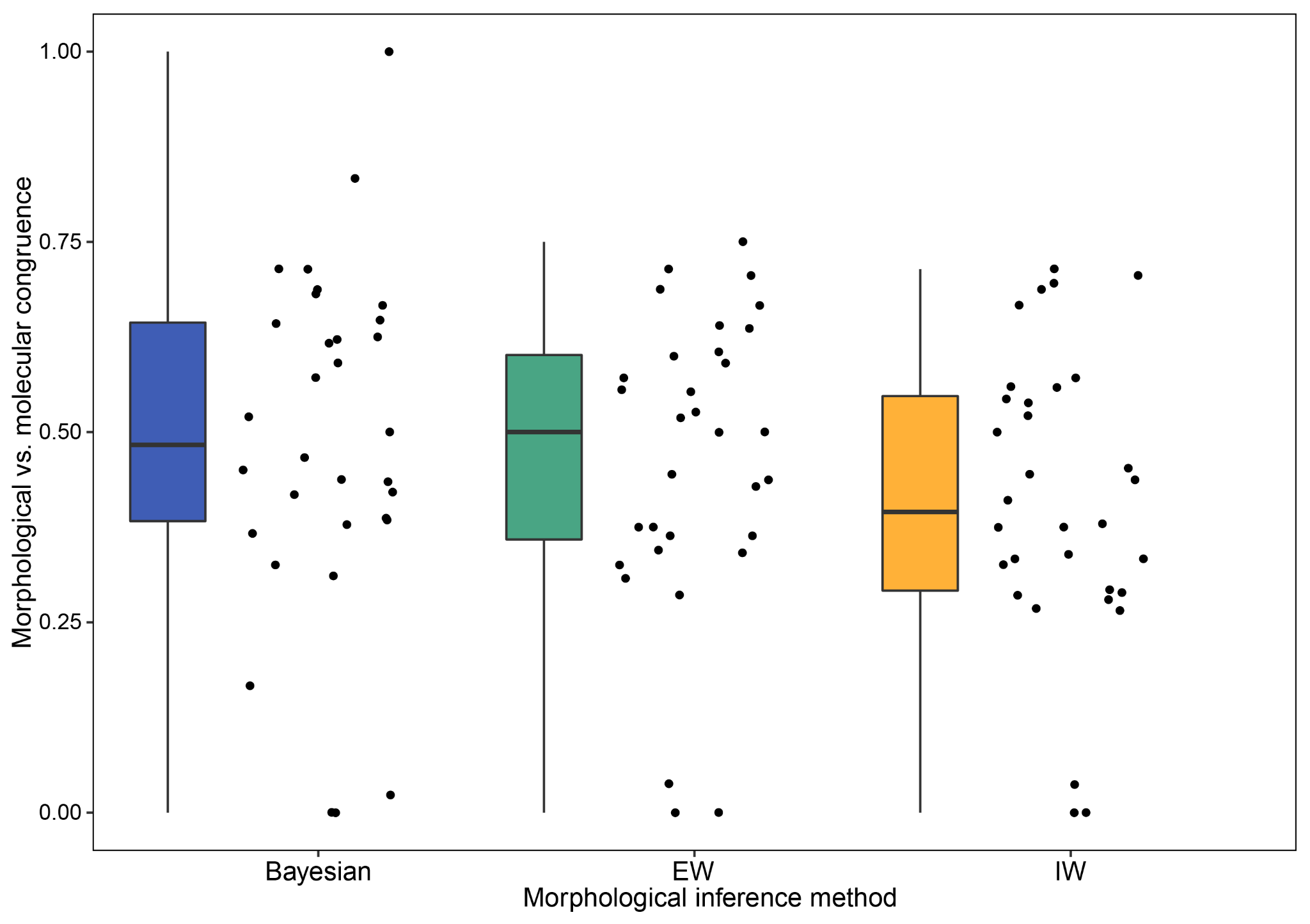


*Supplementary Fig. 2. Parsimony strict consensus trees and Bayesian majority rule consensus trees estimated from morphological data have significantly different congruence with molecular trees (p = 0.012, ANOVA with repeated measures). Congruence is measured using the proportion of bipartition statements shared with the molecular-only maximum clade credibility tree. Points represent proportional congruence of each morphological consensus tree (with the corresponding molecular maximum clade credibility tree) per dataset.*

###
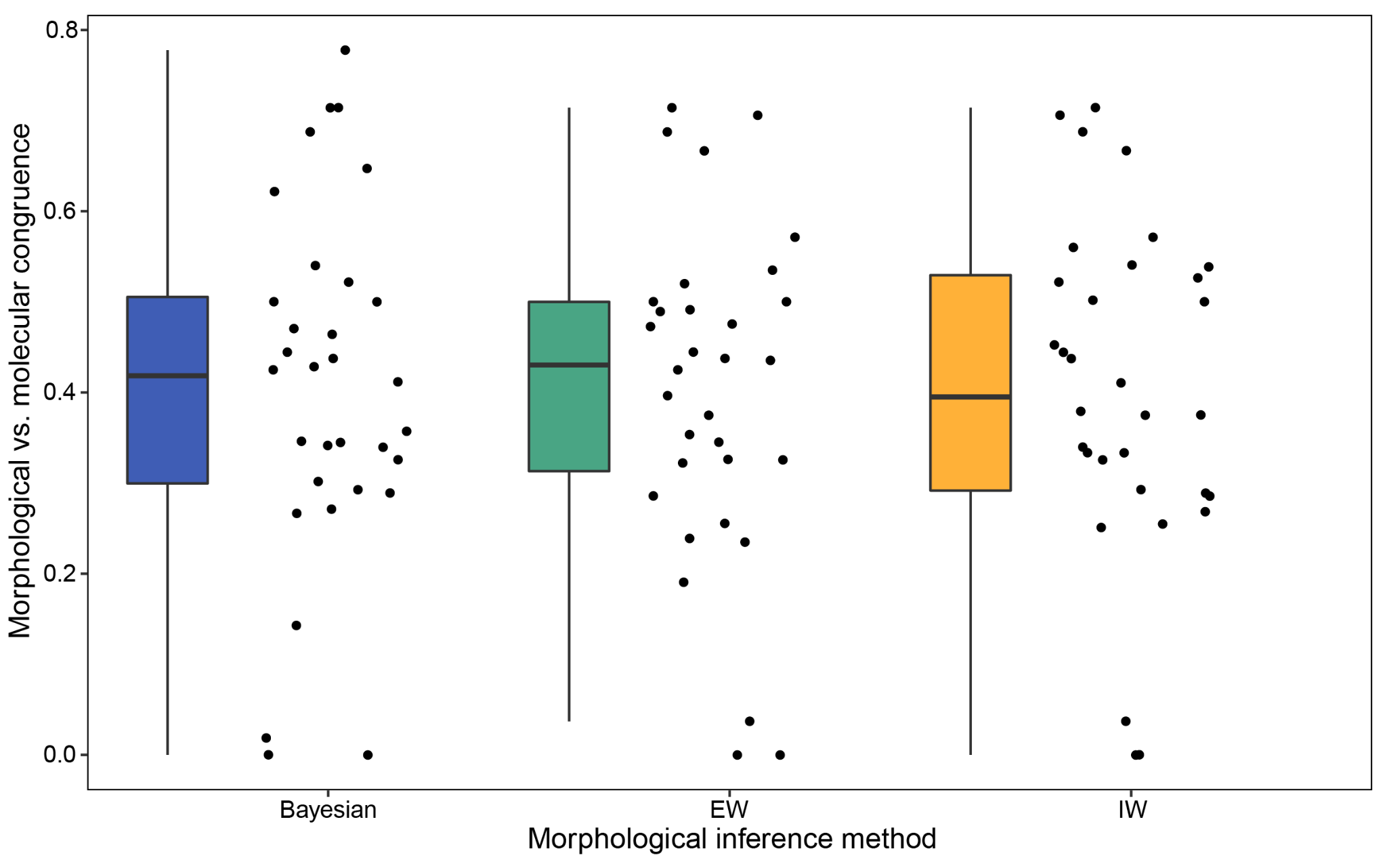


*Supplementary Fig. 3. Optimal morphological trees (i.e. most parsimonious trees and Bayesian maximum clade credibility trees) have similar congruence with the corresponding molecular trees (p = 0.821, ANOVA with repeated measures). Congruence is measured using the mean proportion of bipartition statements that morphological trees share with molecular-only maximum clade credibility tree. The 32 points represent mean proportional congruence between the morphological and molecular trees for each inference method, per dataset.*


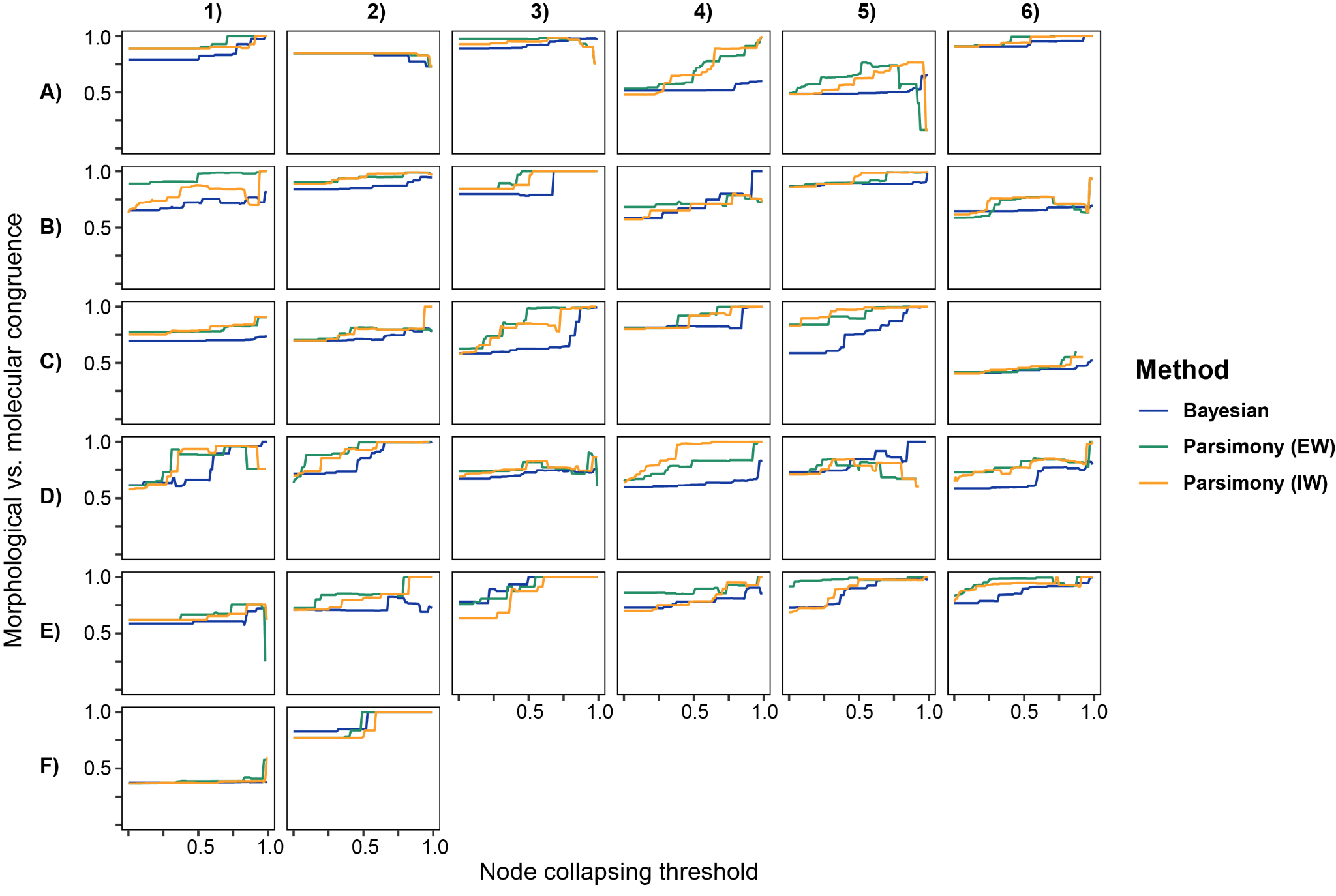


*Supplementary Fig 4. Plot showing the support threshold under which nodes of the morphological consensus tree are collapsed vs. the proportion of quartets in the morphological consensus tree that are congruent with the corresponding molecular maximum clade credibility tree. Panels represent independent empirical total-evidence datasets. Most datasets show a negative correlation between collapsing threshold and proportional congruence; collapsing poorly supported nodes of Bayesian or parsimony morphological trees increases proportional congruence. (A1) Tetraodontiformes (Arcila, D., Pyron, R.A., et al. 2015)*; (A2) *Ostariophysi (Near, T.J., Dornburg, A., et al. 2014); (A3) Mollusca (Vinther, J., Parry, L., et al. 2017); (A4) Mammalia (Lee, M.S. 2016); (A5) Lemuriformes (Herrera, J.P. and Dávalos, L.M. 2016); (A6) Sphenisciformes (Gavryushkina, A., Heath, T.A., et al. 2017) (B1) Hemiptera (Vea, I.M. and Grimaldi, D.A. 2016); (B2) Hymenoptera (Ronquist, F., Klopfstein, S., et al. 2012); (B3) Osteoglossiformes (Lavoué, S. 2016); (B4) Mysticeti (Marx, F.G. and Fordyce, R.E. 2015); (B5) Arthropoda (Lee, M.S., Soubrier, J., et al. 2013); (B6) Squamata (Wiens, J.J., Kuczynski, C.A., et al. 2010); (C1) Serpentes (Harrington, S.M. and Reeder, T.W. 2017); (C2) Palpimanoidea (Wood, H.M., Griswold, C.E., et al. 2012); (C3) Formicidae (Price, S.L., Etienne, R.S., et al. 2016); (C4) Opiliones (Garwood, R.J., Dunlop, J.A., et al. 2011); (C5) Cetecea (Geisler, J.H., McGowen, M.R., et al. 2011); (C6) Malacostraca (Jenner, R.A., Dhubhghaill, C.N., et al. 2009); (D1) Rhynchonellida (Bapst, D.W., Schreiber, H.A., et al. 2018); (D2) Fabriciidae (Huang, D., Fitzhugh, K., et al. 2011); (D3) Stygnopsidae (Cruz-López, J.A. and Francke, O.F. 2017); (D4) Chiroptera (Dávalos, L.M., Velazco, P.M., et al. 2014); (D5) Hydrophilidae (Short, A.E., Cole, J., et al. 2017); (D6) Tribelocephalinae (Forthman, M. and Weirauch, C. 2017); (E1) Apinae (Cameron, S.A. and Mardulyn, P. 2001); (E2) Biblidinae (Garzón‐Orduña, I.J., Marini‐Filho, O., et al. 2013); (E3) Caviidae (Pérez, M.E. and Pol, D. 2012); (E4) Abrotrichini (Teta, P., Cañón, C., et al. 2017); (E5) Hexactinellida (Dohrmann, M., Kelley, C., et al. 2017); (E6) Hydroptilidae (Santos, A.P., Nessimian, J.L., et al. 2016); (F1) Nephilidae (Kuntner, M., Arnedo, M.A., et al. 2013); (F2) Actinopterygii (Giles, S., Xu, G.-H., et al. 2017).*


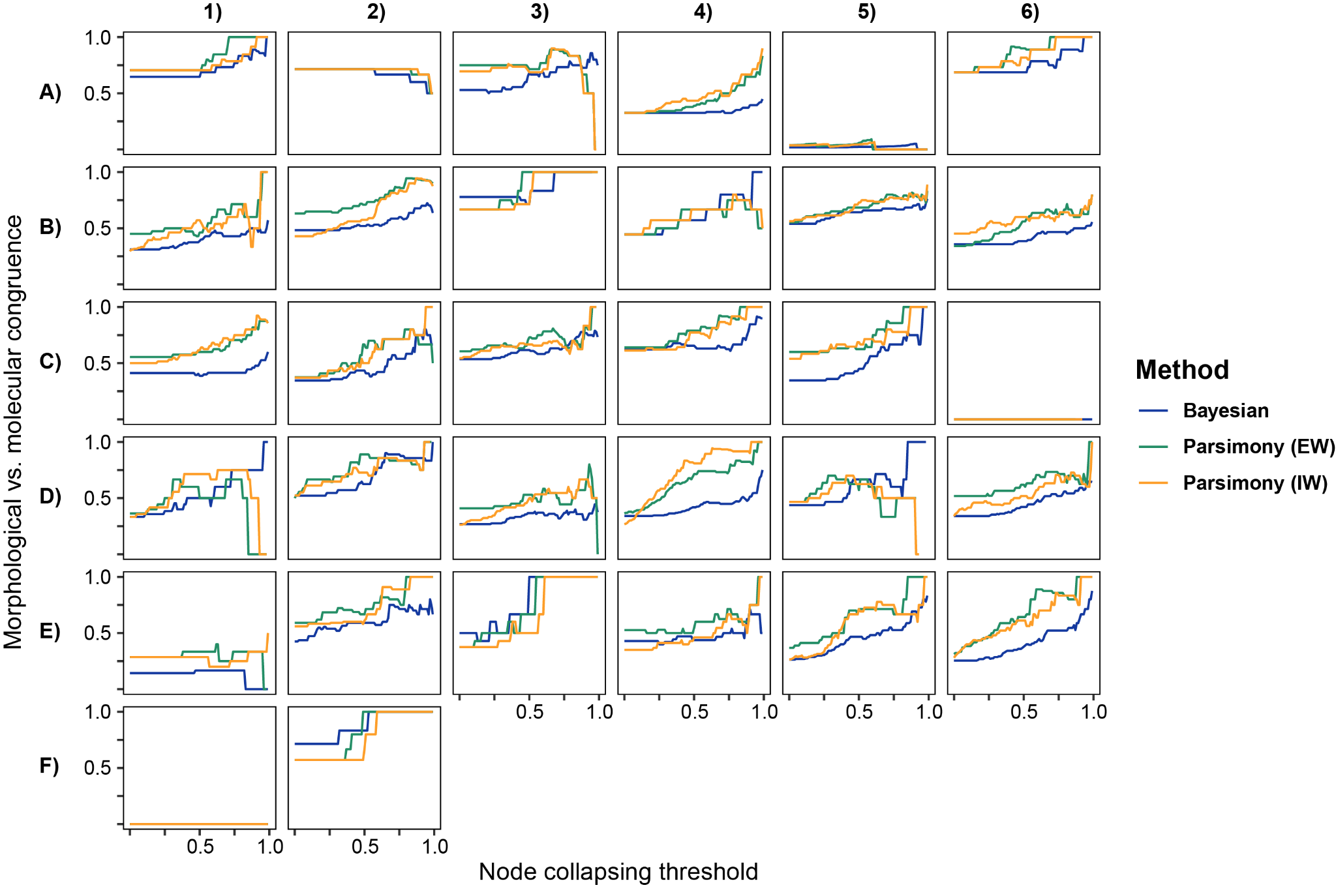
*Supplementary Fig 5. Plot showing the support threshold under which nodes of the morphological consensus tree are collapsed vs. the proportion of bipartitions in the morphological consensus tree that are congruent with the corresponding molecular maximum clade credibility tree. Panels represent independent empirical total-evidence datasets. Most datasets show a negative correlation between collapsing threshold and proportional congruence; collapsing poorly supported nodes of Bayesian or parsimony morphological trees increases proportional congruence. (A1) Tetraodontiformes (Arcila, D., Pyron, R.A., et al. 2015)*; (A2) *Ostariophysi (Near, T.J., Dornburg, A., et al. 2014); (A3) Mollusca (Vinther, J., Parry, L., et al. 2017); (A4) Mammalia (Lee, M.S. 2016); (A5) Lemuriformes (Herrera, J.P. and Dávalos, L.M. 2016); (A6) Sphenisciformes (Gavryushkina, A., Heath, T.A., et al. 2017) (B1) Hemiptera (Vea, I.M. and Grimaldi, D.A. 2016); (B2) Hymenoptera (Ronquist, F., Klopfstein, S., et al. 2012); (B3) Osteoglossiformes (Lavoué, S. 2016); (B4) Mysticeti (Marx, F.G. and Fordyce, R.E. 2015); (B5) Arthropoda (Lee, M.S., Soubrier, J., et al. 2013); (B6) Squamata (Wiens, J.J., Kuczynski, C.A., et al. 2010); (C1) Serpentes (Harrington, S.M. and Reeder, T.W. 2017); (C2) Palpimanoidea (Wood, H.M., Griswold, C.E., et al. 2012); (C3) Formicidae (Price, S.L., Etienne, R.S., et al. 2016); (C4) Opiliones (Garwood, R.J., Dunlop, J.A., et al. 2011); (C5) Cetecea (Geisler, J.H., McGowen, M.R., et al. 2011); (C6) Malacostraca (Jenner, R.A., Dhubhghaill, C.N., et al. 2009); (D1) Rhynchonellida (Bapst, D.W., Schreiber, H.A., et al. 2018); (D2) Fabriciidae (Huang, D., Fitzhugh, K., et al. 2011); (D3) Stygnopsidae (Cruz-López, J.A. and Francke, O.F. 2017); (D4) Chiroptera (Dávalos, L.M., Velazco, P.M., et al. 2014); (D5) Hydrophilidae (Short, A.E., Cole, J., et al. 2017); (D6) Tribelocephalinae (Forthman, M. and Weirauch, C. 2017); (E1) Apinae (Cameron, S.A. and Mardulyn, P. 2001); (E2) Biblidinae (Garzón‐Orduña, I.J., Marini‐Filho, O., et al. 2013); (E3) Caviidae (Pérez, M.E. and Pol, D. 2012); (E4) Abrotrichini (Teta, P., Cañón, C., et al. 2017); (E5) Hexactinellida (Dohrmann, M., Kelley, C., et al. 2017); (E6) Hydroptilidae (Santos, A.P., Nessimian, J.L., et al. 2016); (F1) Nephilidae (Kuntner, M., Arnedo, M.A., et al. 2013); (F2) Actinopterygii (Giles, S., Xu, G.-H., et al. 2017).*

*
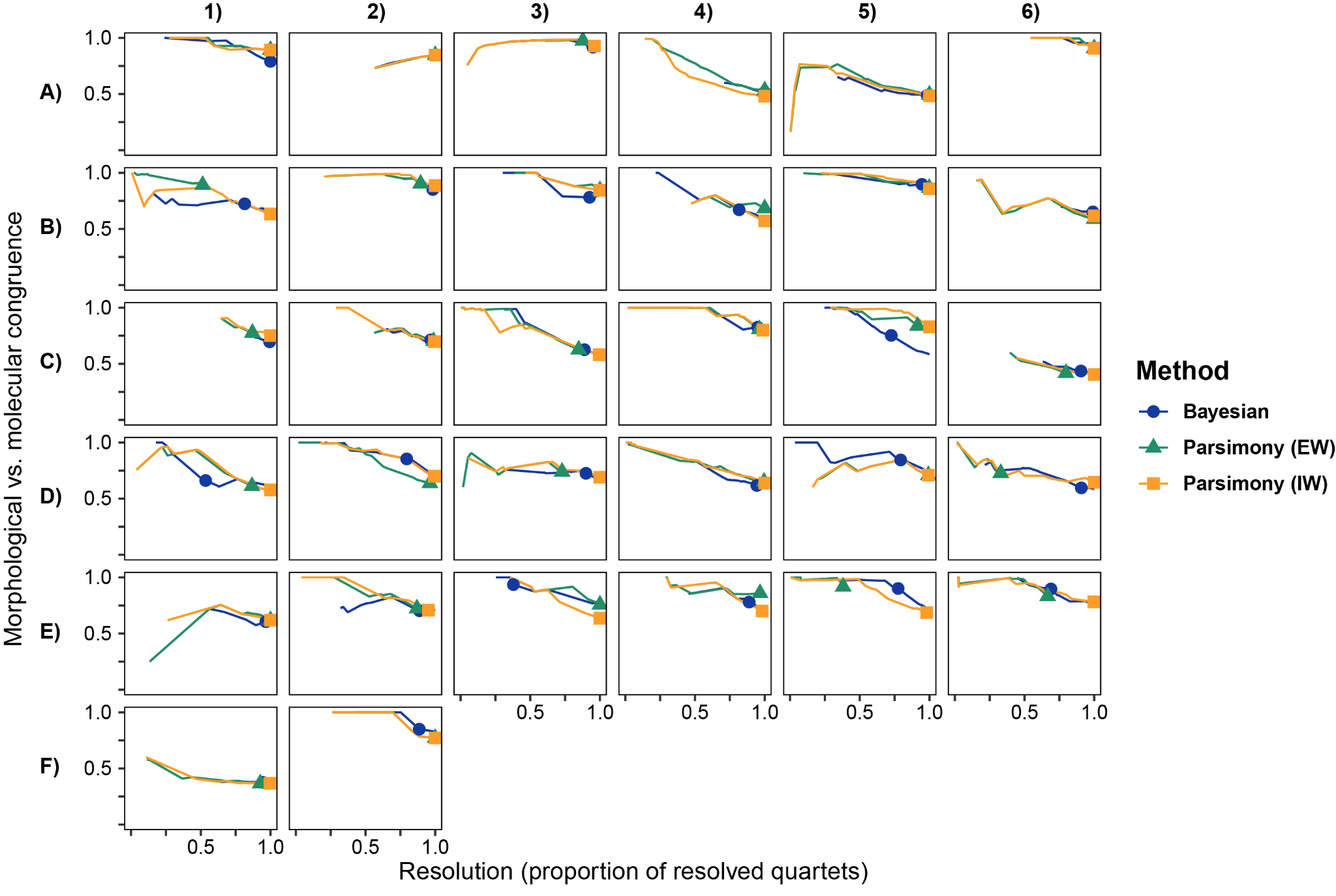
Supplementary Fig 6. Plot showing the proportion of resolved quartets of the morphological consensus tree (as nodes are collapsed iteratively based on support values) vs. the proportion of quartets in the morphological consensus tree that are congruent with the corresponding molecular maximum clade credibility tree. Panels represent independent empirical total-evidence datasets. Points represent the standard consensus trees (Bayesian 50% majority rule consensus, EW strict consensus, IW strict consensus). Bayesian and parsimony trees show similar trajectories as nodes are iteratively collapsed; trees of similar precision show similar congruence. Most datasets show a negative correlation between resolution and proportional congruence. (A1) Tetraodontiformes (Arcila, D., Pyron, R.A., et al. 2015)*; (A2) *Ostariophysi (Near, T.J., Dornburg, A., et al. 2014); (A3) Mollusca (Vinther, J., Parry, L., et al. 2017); (A4) Mammalia (Lee, M.S. 2016); (A5) Lemuriformes (Herrera, J.P. and Dávalos, L.M. 2016); (A6) Sphenisciformes (Gavryushkina, A., Heath, T.A., et al. 2017) (B1) Hemiptera (Vea, I.M. and Grimaldi, D.A. 2016); (B2) Hymenoptera (Ronquist, F., Klopfstein, S., et al. 2012); (B3) Osteoglossiformes (Lavoué, S. 2016); (B4) Mysticeti (Marx, F.G. and Fordyce, R.E. 2015); (B5) Arthropoda (Lee, M.S., Soubrier, J., et al. 2013); (B6) Squamata (Wiens, J.J., Kuczynski, C.A., et al. 2010); (C1) Serpentes (Harrington, S.M. and Reeder, T.W. 2017); (C2) Palpimanoidea (Wood, H.M., Griswold, C.E., et al. 2012); (C3) Formicidae (Price, S.L., Etienne, R.S., et al. 2016); (C4) Opiliones (Garwood, R.J., Dunlop, J.A., et al. 2011); (C5) Cetecea (Geisler, J.H., McGowen, M.R., et al. 2011); (C6) Malacostraca (Jenner, R.A., Dhubhghaill, C.N., et al. 2009); (D1) Rhynchonellida (Bapst, D.W., Schreiber, H.A., et al. 2018); (D2) Fabriciidae (Huang, D., Fitzhugh, K., et al. 2011); (D3) Stygnopsidae (Cruz-López, J.A. and Francke, O.F. 2017); (D4) Chiroptera (Dávalos, L.M., Velazco, P.M., et al. 2014); (D5) Hydrophilidae (Short, A.E., Cole, J., et al. 2017); (D6) Tribelocephalinae (Forthman, M. and Weirauch, C. 2017); (E1) Apinae (Cameron, S.A. and Mardulyn, P. 2001); (E2) Biblidinae (Garzón‐Orduña, I.J., Marini‐Filho, O., et al. 2013); (E3) Caviidae (Pérez, M.E. and Pol, D. 2012); (E4) Abrotrichini (Teta, P., Cañón, C., et al. 2017); (E5) Hexactinellida (Dohrmann, M., Kelley, C., et al. 2017); (E6) Hydroptilidae (Santos, A.P., Nessimian, J.L., et al. 2016); (F1) Nephilidae (Kuntner, M., Arnedo, M.A., et al. 2013); (F2) Actinopterygii (Giles, S., Xu, G.-H., et al. 2017).*


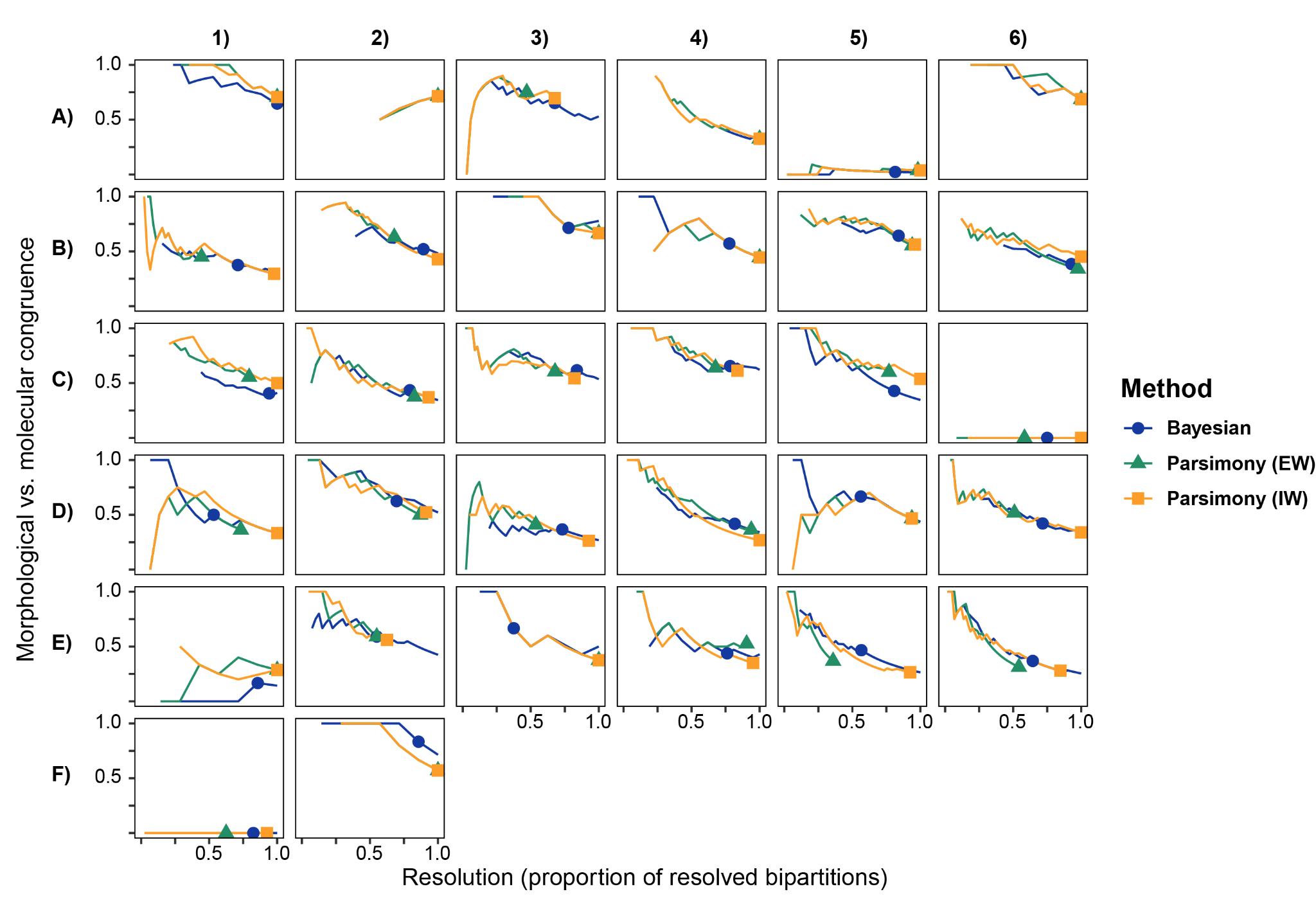
*Supplementary Fig 7. Plot showing the proportion of resolved bipartitions of the morphological consensus tree (as nodes are collapsed iteratively based on support values) vs. the proportion of bipartitions in the morphological consensus tree that are congruent with the corresponding molecular maximum clade credibility tree. Panels represent independent empirical total-evidence datasets. Points represent the standard consensus trees (Bayesian 50% majority rule consensus, EW strict consensus, IW strict consensus). Bayesian and parsimony trees show similar trajectories as nodes are iteratively collapsed; trees of similar precision show similar congruence. Most datasets show a negative correlation between resolution and proportional congruence. (A1) Tetraodontiformes (Arcila, D., Pyron, R.A., et al. 2015)*; (A2) *Ostariophysi (Near, T.J., Dornburg, A., et al. 2014); (A3) Mollusca (Vinther, J., Parry, L., et al. 2017); (A4) Mammalia (Lee, M.S. 2016); (A5) Lemuriformes (Herrera, J.P. and Dávalos, L.M. 2016); (A6) Sphenisciformes (Gavryushkina, A., Heath, T.A., et al. 2017) (B1) Hemiptera (Vea, I.M. and Grimaldi, D.A. 2016); (B2) Hymenoptera (Ronquist, F., Klopfstein, S., et al. 2012); (B3) Osteoglossiformes (Lavoué, S. 2016); (B4) Mysticeti (Marx, F.G. and Fordyce, R.E. 2015); (B5) Arthropoda (Lee, M.S., Soubrier, J., et al. 2013); (B6) Squamata (Wiens, J.J., Kuczynski, C.A., et al. 2010); (C1) Serpentes (Harrington, S.M. and Reeder, T.W. 2017); (C2) Palpimanoidea (Wood, H.M., Griswold, C.E., et al. 2012); (C3) Formicidae (Price, S.L., Etienne, R.S., et al. 2016); (C4) Opiliones (Garwood, R.J., Dunlop, J.A., et al. 2011); (C5) Cetecea (Geisler, J.H., McGowen, M.R., et al. 2011); (C6) Malacostraca (Jenner, R.A., Dhubhghaill, C.N., et al. 2009); (D1) Rhynchonellida (Bapst, D.W., Schreiber, H.A., et al. 2018); (D2) Fabriciidae (Huang, D., Fitzhugh, K., et al. 2011); (D3) Stygnopsidae (Cruz-López, J.A. and Francke, O.F. 2017); (D4) Chiroptera (Dávalos, L.M., Velazco, P.M., et al. 2014); (D5) Hydrophilidae (Short, A.E., Cole, J., et al. 2017); (D6) Tribelocephalinae (Forthman, M. and Weirauch, C. 2017); (E1) Apinae (Cameron, S.A. and Mardulyn, P. 2001); (E2) Biblidinae (Garzón‐Orduña, I.J., Marini‐Filho, O., et al. 2013); (E3) Caviidae (Pérez, M.E. and Pol, D. 2012); (E4) Abrotrichini (Teta, P., Cañón, C., et al. 2017); (E5) Hexactinellida (Dohrmann, M., Kelley, C., et al. 2017); (E6) Hydroptilidae (Santos, A.P., Nessimian, J.L., et al. 2016); (F1) Nephilidae (Kuntner, M., Arnedo, M.A., et al. 2013); (F2) Actinopterygii (Giles, S., Xu, G.-H., et al. 2017).*


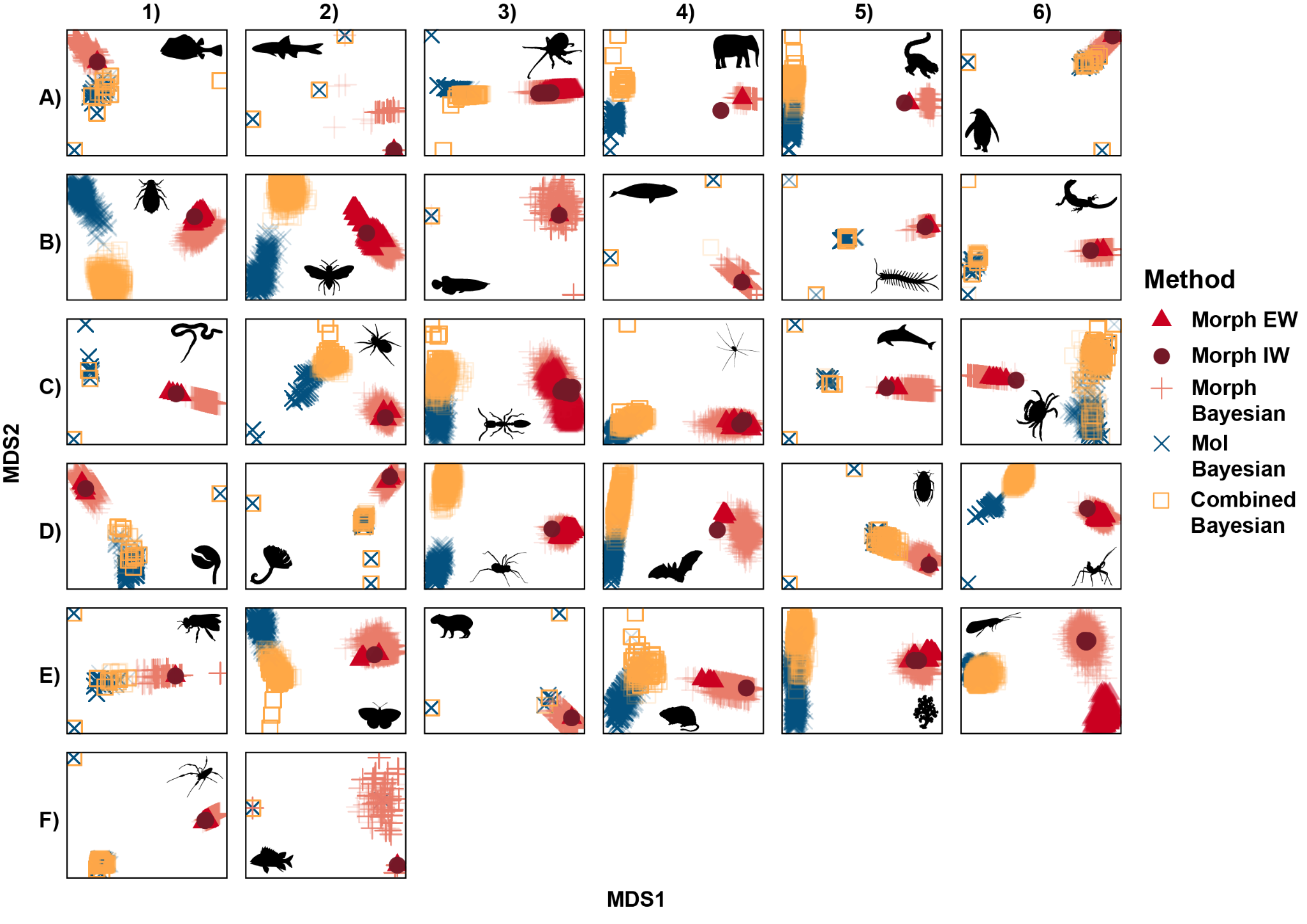
 *Supplementary Fig 8. Treespace visualisation of 32 empirical datasets using the Robinson-Foulds distance metric. Visualizations show Bayesian molecular-only posterior trees (blue crosses); Bayesian combined morphology and molecular posterior trees (open orange squares); Bayesian morphology only posterior trees (pink crosses); equal weighting most parsimonious trees (red triangles) and implied weighting most parsimonious trees (dark red circles). Trees sampled using various morphological methods tend to cluster together; molecular and combined trees tend to be more similar than molecular and morphology trees, but combined trees seldom completely overlap with molecular-only trees; combined analyses thus sample unique areas of treespace. (A1) Tetraodontiformes*; (A2) *Ostariophysi; (A3) Mollusca; (A4) Mammalia; (A5) Lemuriformes; (A6) Sphenisciformes; (B1) Hemiptera; (B2) Hymenoptera; (B3) Osteoglossiformes; (B4) Mysticeti; (B5) Arthropoda; (B6) Squamata; (C1) Serpentes; (C2) Palpimanoidea; (C3) Formicidae; (C4) Opiliones; (C5) Cetacea; (C6) Malacostraca; (D1) Rhynchonellida; (D2) Fabriciidae; (D3) Stygnopsidae; (D4) Chiroptera; (D5) Hydrophilidae; (D6) Tribelocephalinae; (E1) Apinae; (E2) Biblidinae; (E3) Caviidae; (E4) Abrotrichini; (E5) Hexactinellida; (E6) Hydroptilidae; (F1) Nephilidae; (F2) Actinopterygii. Silhouettes for C4, D3 and E6 were created by Gareth Monger, Jennifer Trimble and JCGiron respectively and are reproduced here under the CC BY 3.0 licence.*

| **Clade** | **Reference** | **Ntaxa** | **Char. (morph informative)** | **Char. (mol informative)** | **Char. (morph/all)** | **Unique clades in combined MRC** |
| --- | --- | --- | --- | --- | --- | --- |
| Tetraodontiformes | Arcila et al 2015 | 20 | 185 | 3293 | 0.05 | 2 |
| Ostariophysi | Near et al 2014 | 10 | 76 | 1854 | 0.04 | 0 |
| Mollusca | Vinther et al 2017 | 37 | 76 | 785 | 0.09 | 1 |
| Mammalia | Lee 2016 | 46 | 3414 | 19181 | 0.15 | 4 |
| Lemuriformes | Herrera & Dávalos 2016 | 57 | 346 | 1991 | 0.15 | 5 |
| Sphenisciformes | Gavryushkina et al 2017 | 19 | 75 | 656 | 0.1 | 0 |
| Hemiptera | Vea & Grimaldi 2016 | 48 | 163 | 1394 | 0.1 | 9 |
| Hymenoptera | Ronquist et al 2012 | 59 | 334 | 1847 | 0.15 | 9 |
| Osteoglossiformes | Lavoué 2016 | 12 | 69 | 4969 | 0.01 | 0 |
| Mysticeti | Marx & Fordyce 2015 | 12 | 143 | 2621 | 0.05 | 1 |
| Arthropoda | Lee et al 2013 | 53 | 327 | 6457 | 0.05 | 0 |
| Squamata | Wiens et al 2010 | 45 | 298 | 6010 | 0.05 | 1 |
| Serpentes | Harrington & Reeder 2017 | 37 | 522 | 11011 | 0.05 | 2 |
| Palpimanoidea | Wood et al 2012 | 32 | 117 | 1793 | 0.06 | 6 |
| Formicidae | Price et al 2016 | 59 | 98 | 1188 | 0.08 | 3 |
| Opiliones | Garwood et al 2011 | 40 | 123 | 922 | 0.12 | 1 |
| Cetacea | Geisler et al 2011 | 29 | 271 | 8158 | 0.03 | 0 |
| Malacostraca | Jenner et al 2009 | 15 | 132 | 1620 | 0.08 | 5 |
| Rhynchonellida | Bapst et al 2018 | 18 | 54 | 361 | 0.13 | 2 |
| Fabriciidae | Huang et al 2011 | 26 | 45 | 3107 | 0.01 | 0 |
| Stygnopsidae | Cruz-López & Francke 2017 | 44 | 66 | 1061 | 0.06 | 8 |
| Chiroptera | Dávalos et al 2014 | 85 | 241 | 3232 | 0.07 | 2 |
| Hydrophilidae | Short et al 2017 | 19 | 34 | 348 | 0.09 | 1 |
| Tribelocephalinae | Forthman & Weirauch 2017 | 56 | 123 | 1170 | 0.1 | 7 |
| Apinae | Cameron & Mardulyn 2001 | 10 | 90 | 533 | 0.14 | 0 |
| Biblidinae | Garzón‐Orduñ et al 2013 | 43 | 85 | 740 | 0.1 | 3 |
| Caviidae | Perez & Pol 2012 | 11 | 79 | 716 | 0.1 | 0 |
| Abrotrichini | Teta et al 2017 | 24 | 96 | 549 | 0.15 | 0 |
| Hexactinellida | Dohrmann et al 2017 | 56 | 107 | 1295 | 0.08 | 7 |
| Hydroptilidae | Santos et al 2016 | 62 | 98 | 986 | 0.09 | 8 |
| Nephilidae | Kuntner et al 2013 | 43 | 198 | 1830 | 0.1 | 0 |
| Actinopterygii | Giles et al 2017 | 10 | 112 | 3429 | 0.03 | 0 |

*Supplementary Table 1. Total-evidence datasets used in our study; the number of tips in the modified dataset (i.e. following removal of tips that lack morphological or molecular partitions); the number of parsimony informative morphological characters; the number of parsimony informative molecular characters; and the number of unique clades in the combined data Bayesian majority rule consensus tree that are not observed in the molecular or morphological Bayesian majority rule consensus trees.*

|  | **M1 – unlinked topology** | | | | | | **M2 – linked topology** | | | | | |  | | |
| --- | --- | --- | --- | --- | --- | --- | --- | --- | --- | --- | --- | --- | --- | --- | --- |
| **Dataset** | **run1** | **run2** | **run3** | **run4** | **Mean*** | **Sd**** | **run1** | **run2** | **run3** | **run4** | **Mean*** | **Sd**** | **Converged?** | **BF** | **Interpretation** |
| Tetraodontiformes | -67571.23 | -67571.12 | -67571.14 | -67570.7 | -67571.02 | 0.24 | -67022.86 | -67025.88 | -67022.4 | -67022.23 | -67022.74 | 1.71 | Y | 548.28 | A |
| Ostariophysi | -27264.22 | -27019.99 | -27265.34 | -27264.19 | -27021.38 | 122.3 | -26419.2 | -26734.79 | -26734.8 | -26418.34 | -26419.37 | 182.46 | N | 602.01 | NA |
| Mollusca | -38719.39 | -38720.81 | -38719.93 | -38720.61 | -38720.03 | 0.65 | -38657.64 | -38657.16 | -38656.41 | -38657.8 | -38657.8 | 0.62 | Y | 62.23 | A |
| Mammalia | -515885.03 | -515887.34 | -515887.5 | -515892.13 | -515886.25 | 2.98 | -514171.33 | -515486.62 | -514163.33 | -514171.63 | -514164.71 | 589.83 | N | 1721.54 | NA |
| Lemuriformes | -60328.45 | -60326.98 | -60327.02 | -60327.35 | -60327.31 | 0.69 | -62013.37 | -62014.83 | -62014.48 | -62014.57 | -62014.14 | 0.65 | Y | -1686.83 | F |
| Sphenisciformes | -18429.35 | -18429.04 | -18429.41 | -18237.29 | -18238.68 | 95.99 | -17662.19 | -17662.32 | -17662.32 | -17662.31 | -17662.24 | 0.06 | N | 576.44 | NA |
| Hemiptera | -37631.32 | -37631.44 | -37631.38 | -37632.3 | -37631.54 | 0.46 | -37410.25 | -37412.93 | -37561.38 | -37411.86 | -37411.39 | 74.86 | N | 220.15 | NA |
| Hymenoptera | -59386.48 | -59384.77 | -59384.35 | -59386.19 | -59385.08 | 1.05 | -52972.44 | -48765.55 | -42776.22 | -51127.8 | -42777.6 | 4437.2 | N | 0 | NA |
| Osteoglossiformes | -97396.03 | -97397.01 | -97396.22 | -97396.39 | -97396.35 | 0.42 | -97025.62 | -97026.03 | -97026.23 | -97024.99 | -97025.6 | 0.55 | Y | 370.75 | A |
| Mysticeti | -92828.1 | -92827.26 | -92827.62 | -92827.06 | -92827.44 | 0.46 | -92831.48 | -92831.72 | -92831.8 | -92832.57 | -92831.82 | 0.47 | Y | -4.38 | E |
| Arthropoda | -219754.83 | -219750.72 | -219756.98 | -219750.44 | -219751.25 | 3.2 | -219695.74 | -219698.07 | -219694.5 | -219695.42 | -219695.34 | 1.52 | Y | 55.91 | A |
| Squamata | -162883.08 | -163623.66 | -163377.31 | -163660.31 | -162884.47 | 358.11 | -163872.47 | -163823.38 | -163755.6 | -163842.29 | -163756.98 | 49.54 | N | -872.51 | NA |
| Serpentes | -246509.87 | -246511.84 | -246514.57 | -246506.36 | -246507.71 | 3.45 | -246621.14 | -246622.93 | -246620.02 | -246623.05 | -246621.05 | 1.47 | Y | -113.34 | F |
| Palpimanoidea | -45259.94 | -45259.44 | -45258.67 | -45259.84 | -45259.34 | 0.58 | -45223.74 | -45223.28 | -45223.84 | -45223.31 | -45223.51 | 0.29 | Y | 35.83 | A |
| Formicidae | -41525.69 | -41528.04 | -41526.47 | -41527.43 | -41526.53 | 1.04 | -41423.95 | -41426.77 | -41427.32 | -41425.86 | -41425.12 | 1.48 | Y | 100.67 | A |
| Opiliones | -24393.43 | -24393.77 | -24393.96 | -24393.03 | -24393.48 | 0.41 | -24308.62 | -24307.37 | -24308.73 | -24310.12 | -24308.28 | 1.12 | Y | 85.2 | A |
| Cetacea | -253992.91 | -253696.38 | -254002.93 | -253791.38 | -253697.77 | 151.77 | -254013.19 | -253855.29 | -254002.19 | -254013.85 | -253856.68 | 77.41 | N | -158.91 | NA |
| Malacostraca | -23039.75 | -23040.04 | -23040.04 | -23040.79 | -23040.15 | 0.44 | -23067.85 | -23069 | -23068.43 | -23068.31 | -23068.32 | 0.47 | Y | -28.17 | F |
| Rhynchonellida | -10989.67 | -10989.5 | -10989.58 | -10989.47 | -10989.55 | 0.09 | -10987.33 | -10987.38 | -10986.93 | -10987.3 | -10987.22 | 0.21 | Y | 2.33 | C |
| Fabriciidae | -26878.38 | -26878.34 | -26876.14 | -26878.17 | -26877.23 | 1.08 | -26465.62 | -26464.92 | -26465.46 | -26464.86 | -26465.16 | 0.38 | Y | 412.07 | A |
| Stygnopsidae | -37341.71 | -37342.03 | -37342.01 | -37342.15 | -37341.96 | 0.19 | -37447.92 | -37448.58 | -37447.14 | -37448.11 | -37447.8 | 0.6 | Y | -105.84 | F |
| Chiroptera | -112424.79 | -112423.17 | -112425.27 | -112427.23 | -112424.26 | 1.67 | -113409.73 | -113449.32 | -113378.11 | -113323.01 | -113324.39 | 53.35 | N | -900.13 | NA |
| Hydrophilidae | -11777.31 | -11777.72 | -11777.49 | -11777.33 | -11777.45 | 0.19 | -11759.46 | -11759.87 | -11759.65 | -11759.99 | -11759.72 | 0.24 | Y | 17.73 | A |
| Tribelocephalinae | -40204.79 | -40067.4 | -40080.93 | -39913.16 | -39914.55 | 119.51 | -40218.95 | -40203.74 | -40077.8 | -40175.8 | -39825.85 | 63.42 | N | 88.7 | NA |
| Apinae | -10847.98 | -10847.38 | -10847.32 | -10847.98 | -10847.61 | 0.36 | -10613.52 | -10860.06 | -10859.98 | -10860.11 | -10614.91 | 123.27 | N | 232.7 | NA |
| Biblidinae | -19262.92 | -19262.71 | -19260.84 | -19262.27 | -19261.81 | 0.94 | -19147.96 | -19147.37 | -19147.35 | -19146.3 | -19147.05 | 0.69 | Y | 114.76 | A |
| Caviidae | -16785.06 | -16784.95 | -16786.13 | -16965.87 | -16785.55 | 90.25 | -16773.98 | -16773.87 | -16774.72 | -16774.85 | -16774.26 | 0.5 | N | 11.29 | NA |
| Abrotrichini | -20137.69 | -20137.09 | -20137.97 | -20137.98 | -20137.61 | 0.42 | -20113.42 | -20113.61 | -20113.5 | -20113.64 | -20113.54 | 0.1 | Y | 24.07 | A |
| Hexactinellida | -38466.29 | -38465.53 | -38462.34 | -38463.99 | -38463.5 | 1.75 | -38302.17 | -38302.63 | -38301.95 | -38301.78 | -38302.09 | 0.37 | Y | 161.41 | A |
| Hydroptilidae | -36555.31 | -36550.89 | -36800.57 | -36730.53 | -36552.26 | 119.13 | -37043.37 | -37043.85 | -37042.89 | -37120.58 | -37043.58 | 38.61 | N | -20258.03 | NA |
| Nephilidae | -56365.02 | -56366.81 | -56369.09 | -56366.71 | -56366.09 | 1.67 | -57435.69 | -57446.11 | -57444.86 | -57443.19 | -57437.08 | 4.67 | Y | -1070.99 | F |
| Actinopterygii | -53439.2 | -53442.28 | -53436.79 | -53443.67 | -53438.09 | 3.09 | -53435.34 | -53434.87 | -53427.74 | -53431.61 | -53429.1 | 3.52 | Y | 8.99 | A |

*Supplementary Table 2. Results of stepping stone Bayes factor analysis. Interpretations: A) Bayes Factor > 5 = very strong evidence datasets are combinable; B) Bayes Factor > 3 & < 5 = strong evidence datasets are combinable; C) Bayes Factor > 0 & < 3 = weak evidence datasets are combinable; D) Bayes Factor < 0 & > -3 = weak evidence datasets are uncombinable; E) Bayes Factor < -3 & > -5 = strong evidence datasets are uncombinable; F) Bayes Factor < -5 = very strong evidence datasets are uncombinable. * Mean marginal likelihood calculated by MrBayes following stepping stone analysis. **SD of marginal likelihood estimates from indepndent runs of MrBayes stepping stone analysis.*
